# From support to pressure: the multiple associated effects of differential leadership patterns on the workplace behavior of university teachers in western China

**DOI:** 10.3389/fpsyg.2026.1855518

**Published:** 2026-05-26

**Authors:** Junfeng Si, Yuan Xun, Nannan Ye, Junxuan Li, Yuguang Tang

**Affiliations:** 1 College of International Education, Sichuan International Studies University, Chongqing, China; 2 Institute of Higher Education, East China Normal University, Shanghai, China; 3 Guangdong Education Publishing House, Guangzhou, China; 4 Department of Educational Psychology, The Chinese University of Hong Kong, Shatin, Hong Kong SAR, China

**Keywords:** differential leadership, job burnout, professional identity, psychological capital, social comparison, university teachers in western China

## Abstract

**Introduction:**

Although differential leadership is prevalent in Chinese organizations, its multiple effects on the workplace well-being and behavior of university teachers remain under-explored. Drawing on social comparison and professional identity theories, this study investigates how differential leadership predicts job performance, turnover intention, and burnout among western Chinese university teachers.

**Methods:**

This study utilized a complementary design: a scenario experiment (Study 1, *N* = 752) manipulating “ingroup-outgroup” relational cues to capture immediate psychological reactions, and a cross-sectional survey (Study 2, *N* = 713) across 22 universities to assess real-world organizational perceptions and associational patterns. Study 1 was designed to provide evidence on the immediate responses triggered by relational cues, whereas Study 2 was used to examine naturally occurring relationships without claiming strict causality.

**Findings:**

The results revealed a consistent serial indirect path through social comparison and professional identity. Differential leadership positively predicted social comparison, which in turn diminished professional identity, subsequently relating to lower job performance and higher turnover intention and burnout. Furthermore, psychological capital served as an important boundary condition in Study 2. Notably, the two-study pattern suggests a possible temporal heterogeneity: sudden “outgroup exclusion” evoked immediate psychological withdrawal where the moderating role of psychological capital was not significant in Study 1, whereas the buffering association involving psychological capital was statistically evident in Study 2’s cross-sectional field data.

**Discussion:**

The findings expand the boundaries of localized leadership theories in higher education by demonstrating that the impacts of differential leadership on teachers’ workplace outcomes appear to vary across relational positions and time scales. However, because Study 2 used cross-sectional self-report data, the long-term mechanism should be interpreted as a correlational rather than causal process. The study provides valuable empirical guidance for enhancing university governance transparency to mitigate disorganized comparison pressures.

## 1 Introduction

In academic organizations, pressure does not always stem from standard task loads. While teaching and research constitute a high-intensity work rhythm, professional exhaustion often transcends physical labor, frequently stemming from perceived disparities in access to trust, resources, and informal support.

This phenomenon is rooted in the cultural logic of the differential mode of association (chaxugeju), differential leadership (Liu N. et al., 2023; Liu Y. et al., 2023; Liu T. et al., 2024; Liu Y. et al., 2024; Wang et al., 2025; Jiang et al., 2023) precisely describes a management model based on relationship closeness, emotional connection, and trust. Unlike general empowerment, it involves a stratified structure where members are positioned at the organizational center and others at the periphery, leading to either developmental support or opaque biases and relational barriers. Research on broader leadership dynamics suggests that leadership agency and its distributed structures are fundamental in fostering organizational trust and commitment, which are pivotal for positive work outcomes (Barattucci et al., 2020).

The academic context further complicates this phenomenon. Theoretically, universities should emphasize resource allocation based on academic standards; however, informal support networks are equally active. Access to information, platform recommendations, and administrative encouragement are often influenced by these informal ties rather than institutional texts alone. The context of universities in western China further amplifies this issue because resource scarcity intensifies teachers’ sensitivity toward relational-based distribution.

The effects of differential leadership are dual-sided. While “ingroup” members may benefit from trust and visibility, “outgroup” members often experience comparison pressure and depletion. Thus, differential leadership is not merely a single evaluative management label; rather, it acts as an organizational relationship structure, and its outputs depend on the relational position an individual occupies within this structure and on the transparency of rule explanations.

Instead of assuming purely negative outcomes, this study utilizes dual evidence from experiments and surveys to examine whether social comparison and professional identity (Cai et al., 2024) explain the divergent effects. Furthermore, it explores whether psychological capital serves as a critical buffer in these associations (Hazan-Liran and Karni-Vizer, 2024; Xue et al., 2023).

To explore these dynamics, this paper conducts verification through two studies. Study 1 uses a scenario experiment to capture the short-term responses of differential support and differential exclusion; Study 2 employs a field survey to examine these associations in a real-world organizational context. This study contributes to the literature by conceptualizing differential leadership as a dual-edged organizational structure. By integrating social comparison and professional identity mechanisms, it reveals how relational distance translates into divergent behavioral responses, providing empirical guidance for university governance in localized contexts.

## 2 Theoretical background and research hypotheses

### 2.1 The local connotation of differential leadership and its applicability in universities

Differential leadership is not a standardized leadership behavior concept in Western organizational theory, but rather a localized construct rooted in Chinese social structure and relationship culture (Jiang et al., 2023; Liu et al., 2026). Rather than the leader’s institutional management treating all subordinates equally, or differentiated empowerment entirely based on performance, it involves a stratified leadership style influenced by relational closeness, emotional identification, and historical interaction. In this pattern, organizational members form different levels of “near-far structures” around the leader. For those close to the center, this structure means trust, support, and development opportunities; for peripheral members, it may mean information lag, resource dilution, and relative deprivation. This difference in psychological perception caused by relational positions underpins the differentiated phenomena differential leadership produces on subordinates. Prior research has shown that differential leadership is closely related to employees’ in-role performance (Chen and Haga, 2022).

Differential leadership in university organizations merits special discussion because universities do not operate entirely on rigid processes. Teaching, research, and administrative work are often intertwined, and many key resources flow under the joint action of formal systems and informal support networks. For teachers, the impact is not only the “gain and loss” at the outcome level but also the basis for judging organizational justice and career predictability.

### 2.2 How relational differences translate into social comparison

Social comparison theory (Caliskan et al., 2024; Gibbons and Buunk, 1999) points out that when individuals lack stable self-evaluation standards, they rely on the situations and performance of others to understand themselves. In universities, this process is particularly common as work achievements are usually delayed and cumulative; making it difficult to receive immediate feedback on research and teaching outputs. This means that individuals view the resource acquisitions and opportunity accessibility of surrounding colleagues as important benchmarks for judging their own positions.

Differential leadership creates a comparison environment that can be continuously observed. Visibile cues of differential treatment—such as prioritized core projects or symbolic recognition**—**are highly visible to teachers. In western universities where resources are limited, this visibility is often amplified, as resource scarcity intensifies the focus on distribution methods.

Consequently, differential leadership prompts teachers to enter a state of comparison. From the perspective of situational effects, when teachers perceive themselves at the organizational periphery (outgroup), these contrast cues will directly relate to an upward social comparison tendency (Cao et al., 2025). Although social comparison may take multiple forms, the present study focuses on the overall comparison tendency. In the context of differential leadership, upward comparison with better-supported colleagues is especially salient for outgroup teachers. This situational tendency (Study 1) and relational perception (Study 2) are logically consistent, both manifesting as a positive association between differential perception and social comparison.

*H1*: Differential leadership perception positively predicts teachers’ social comparison tendencies.

### 2.3 The erosive effect of social comparison on professional identity

Social comparison revolving around relational bias rather than academic merit is likely to trigger a sense of imbalance. As individuals observe the “relational distance” between others and themselves, they re-evaluat their own professional value and organizational significance.

Professional identity is the degree to which teachers internalize their professional roles as an important component of their self-concept (Halal Orfali et al., 2024; Uras Eren and Atay, 2025). When teachers find themselves in disadvantageous comparisons, the positive meaning of the profession declines. On the one hand, constant upward comparison may cause teachers to interpret resource gaps as “I am not valued” (Cai et al., 2024). This doubt will slowly weaken the connection between the professional role and self-value.

*H2*: Social comparison is negatively associated with professional identity.

### 2.4 Professional identity and teachers’ behavioral performance

Professional identity manifests at the behavioral level. For teachers, higher professional identity usually means stronger teaching engagement and a higher degree of organizational integration. Conversely, if an individual’s sense of value in the teacher role declines, behavioral withdrawal may follow. This withdrawal can be reflected in diminished work enthusiasm, thoughts of leaving (You and Li, 2023), and emotional exhaustion (Maslach and Jackson, 1981; Maslach et al., 2001).

University work relies on endogenous motivation. Teachers need to maintain long-term investment in affairs that cannot be fully externally supervised (e.g., student guidance and research revision). Professional identity is the resource that maintains the continuity of teachers’ behaviors. When this resource is weakened, declines in performance, rising turnover intentions, and accumulation of burnout emerge.

*H3a*: Professional identity positively relates to job performance.

*H3b*: Professional identity is negatively linked to turnover intention.

*H3c*: Professional identity corresponds to lower job burnout.

### 2.5 From relational perception to behavioral outcomes: the serial mediation logic

If differential leadership predicts social comparison, and social comparison weakens professional identity, then a psychological chain may exist between differential leadership and teachers’ behavioral outcomes. This serial path is empirically plausible. Teachers rarely resign immediately due to a single resource allocation difference, rather, repeated differentiation cues foster sustained comparison, gradually weakening the professional role, ultimately resulting in behavioral withdrawal. This path describes a theoretically proposed “cognition—identity—behavior” chain for teachers when dealing with differentiated management.

In summary, the comparison pressure and identity erosion accompanying differential leadership constitute a “cognition—identity—behavior” logical chain.

*H4a*: Social comparison and professional identity constitute a indirect path linking differential leadership and job performance.

*H4b*: Social comparison and professional identity constitute a indirect path linking differential leadership and turnover intention.

*H4c*: Social comparison and professional identity constitute a indirect path linking differential leadership and job burnout.

### 2.6 Psychological capital as a boundary condition

Psychological capital (Luthans et al., 2007) may help individuals understand stressful events in a more constructive way and maintain psychological stability (Hazan-Liran and Karni-Vizer, 2024; Qiu and Shen, 2025; Zhang and Li, 2025). Theoretically, teachers with higher psychological capital may not completely translate differential cues into negative comparisons, viewing temporary disadvantageous situations as phased fluctuations rather than denials of self-worth.

However, the role of psychological capital may possess temporal and situational heterogeneity. Faced with sudden strong relational exclusion, individuals may momentarily generate instinctive stress comparisons, at which time the defensive barrier of trait psychological capital may be briefly weakened. In contrast, for chronic differential structures, psychological capital may exert a more stable buffering role.

*H5*: In normalized organizational contexts, psychological capital significantly attenuates the positive relationship between differential leadership and social comparison.

*H6*: In normalized organizational contexts, psychological capital can further alter the overall strength of the serial mediation by influencing the “differential leadership—social comparison” path.

**Figure 1:**
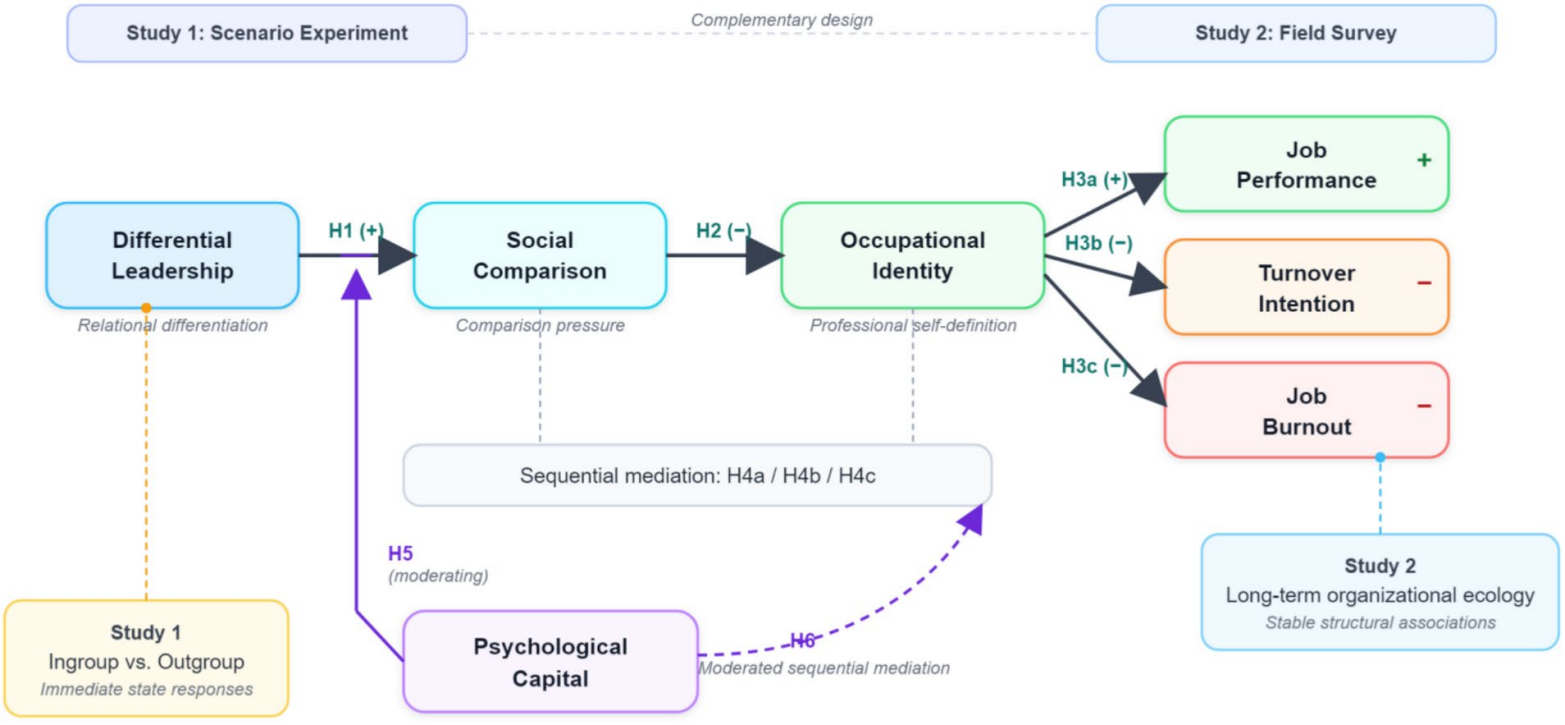
Theoretical model. Solid arrows indicate direct association paths, dashed arrows indicate moderating paths, and “+” and “−” in parentheses indicate hypothesized positive and negative relationships, respectively. Study 1 (scenario experiment) and study 2 (organizational questionnaire), as complementary research designs, correspond to the immediate reaction level and long-term structural level of this model, respectively.

Figure 1 presents the integrated “cognition–identity–behavior” model derived from these hypotheses, framing differential leadership as an antecedent to teachers’ behavioral outcomes through the serial mediation of social comparison and professional identity, with psychological capital as a moderator. The research utilizes a complementary approach: Study 1 assesses immediate reactions to relational cues; whereas Study 2 examines structural patterns within the university ecology.

## 3 Research design and methods

### 3.1 Overall design approach

To analyze the dual effects of differential leadership on university teachers in western China, this study adopts a complementary design combining situational induction and field surveys.

Study 1 utilizes a scenario experiment to examine immediate psychological and behavioral responses to “ingroup–outgroup” relational cues under controlled conditions. This design captures the short-term implications of relational inclusion and exclusion while minimizing complex confounding factors. Study 2 employs a cross-sectional field survey to examine the stable associations among differential leadership perception, social comparison, professional identity, and workplace outcomes. Within real university ecologies. Together, the two studies offer a more comprehensive understanding of how differential leadership is experienced as either support or pressure, balancing internal and ecological validity.

### 3.2 Study 1: scenario experiment

#### 3.2.1 Subjects and procedures

Participants in Study 1 were recruited through Credamo, a professional academic survey platform in China. Screening criteria were used to include only currently employed faculty members from higher education institutions located in western China. Therefore, the recruitment strategy was a platform-based convenience sampling procedure rather than probability sampling. After excluding invalid responses, 752 valid samples were retained, yielding a valid response rate of 91.7%.

The study adopted a single-factor between-subjects design with relational position manipulated as either ingroup or outgroup. Participants were randomly assigned to each condition. Specifically, 395 participants (52.5%) were assigned to the “ingroup” scenario, and 357 participants (47.5%) were assigned to the “outgroup” scenario.

Initial demographic data collection (Table 1) covered a diverse range of genders, ages, titles, providing a basis for subsequent between-group homogeneity testing and analysis of immediate scenario effects. However, the high proportion of doctorate holders suggests that generalizability to the broader population should be interpreted with caution.

**Table 1:** Demographic characteristics of Study 1 sample (*N* = 752).

Variable	Category	Frequency	Percentage (%)
Gender	Male	411	54.7
	Female	329	43.8
	Other	12	1.6
Age	≤30	150	19.9
	31–40	266	35.4
	41–50	219	29.1
	≥51	117	15.6
Education	Doctorate	529	70.3
	Master’s	172	22.9
	Bachelor’s	51	6.8
Title	Teaching Assistant/Lecturer	129	17.2
	Associate Professor	315	41.9
	Professor	235	31.2
	Other	73	9.7
Experimental group	Outgroup	357	47.5
	Ingroup	395	52.5

#### 3.2.2 Manipulation scheme design

The experimental materials were adapted from classical paradigms to the organizational context of Chinese universities. The manipulation constructed two contrasting relational-position cues: relational inclusion and relational exclusion, involving resource allocation, academic recommendations, and informal information access.

The manipulation focused on the most salient signals of differential treatment—positioning the teacher as either a trusted “ingroup” member or a marginalized “outgroup” member—rather than reproducing the full multidimensional structure of the construct.

In contrast, the ingroup scenario portrayed a high-quality exchange relationship where the participant acts as a core backbone of the department, enjoying significant prior advantages and leadership care in major project applications, informal information acquisition, and resource guarantees.

In contrast, the outgroup scenario simulated a administrative state defined by a formal contractual relationship, with significant disadvantages in career procedural support and decision-making inclusion.

Through this contrast, Study 1 examined whether being placed in different relational positions would induce different immediate levels of social comparison, professional identity, and behavioral tendencies.

#### 3.2.3 Variable measurement and description

The measurements mainly included baseline trait variables and state-based tendencies activated during the intervention phase. All items were scored using a 5-point Likert scale.

*Baseline trait variables*: PCQ scale developed by Luthans et al. (2007) was used to measure individuals’ trait psychological capital (12 items) capturing participants’ psychological resource endowment before receiving the experimental manipulation and serving as a moderating benchmark for subsequent interaction analysis.

*Manipulation check*: a single item (“In the above scenario, I think I belong to the people Dean Zhang is relatively close to and trusts”) verified whether participants perceived the intended relational proximity.

*State-based mediators and behavioral tendencies*: core items from authoritative scales were adapted with hypothetical qualifiers (e.g., “In this situation, I would…”) to evaluate: Specifically, it included: State Social Comparison [4 items, adapted from Gibbons and Buunk (1999)]; State Professional Identity [3 items, adapted from Wei et al. (2013)]; and immediate behavioral tendencies including job performance, turnover intention tendency, and job burnout tendency [4 items, adapted from Williams and Anderson (1991)], Turnover Intention [3 items, adapted from Mobley (1977)], and Emotional Exhaustion Feelings [3 items, adapted from Maslach and Jackson (1981)].

#### 3.2.4 Data analysis strategy

Statistical analysis (SPSS 26.0) included Cronbach’s *α* for reliability, independent samples *t*-tests for manipulation checks and group comparisons, and Cohen’s d for effect size evaluation.

Bootstrapping analyses explored the psychological linkage between experimental conditions and behavioral tendencies, while interaction analyses tested the moderating role of psychological capital. Given the cross-sectional nature of these post-scenario sessions, results indicate psychological associations rather than temporal causality.

### 3.3 Study 2: questionnaire survey

#### 3.3.1 Sample source and organizational background

Study 2 surveyed 22 universities across seven western provinces (regions), collecting 750 questionnaires. After strict data cleaning, excluding samples with excessively strong answering patterns, excessive missing values, and duplicate responses, 713 valid questionnaires were retained (valid recovery rate of 95.1%). The sample (Table 2) showed significant variation in relational proximity to leaders (31.4% intimate vs. 68.6% distant), providing a robust context for analysis.

**Table 2:** Demographic characteristics of Study 2 sample (*N* = 713).

Variable	Category	Frequency	Percentage (%)
Gender	Male	346	48.5
	Female	341	47.8
	Other	26	3.6
Age	≤30	122	17.1
	31–40	261	36.6
	41–50	197	27.6
	≥51	133	18.7
Education	Doctorate	512	71.8
	Master’s	173	24.3
	Bachelor’s	28	3.9
Title	Teaching Assistant/Lecturer	97	13.6
	Associate Professor	333	46.7
	Professor	213	29.9
	Other	70	9.8
Perception of relationship with leader	Distant/Relatively Distant	215	30.2
	Average	274	38.4
	Intimate/Relatively Intimate	224	31.4

Study 2 focused on naturally occurring perceptions embedded in teachers’ daily experience, examining stable associations rather than making causal claims.

#### 3.3.2 Measurement tools

Validated 5-point Likert scales (except for job burnout frequency) were used to evaluate variable relationships within their natural organizational ecologies.

*Differential leadership (L)*: differential leadership was measured using Jiang and Zhang (2010) 15-item scale. It contains three dimensions: partiality (6 items), care and communication (5 items), and tolerance for mistakes (4 items), the scale evaluates perceptions of widespread differentiated treatment or relationally biased leadership within the department. Higher scores indicate stronger perceived relational bias rather than greater personal support received from the leader.

*Psychological Capital (PC)*: individual psychological resources were assessed via the PCQ-12 developed by Luthans et al. (2007). It covers four dimensions: self-efficacy, hope, resilience, and optimism, reflecting teachers’ resource reserves in the current work environment.

*Social comparison (SC)*: the 8-item INCOM scale Gibbons and Buunk (1999) was used to measure the tendency to compare one’s professional treatment with that of colleagues. Because the INCOM scale captures general social comparison orientation, the analysis focuses on teachers’ overall comparison frequency rather than specific directional comparisons.

*Professional identity (OI)*: adopted the University Teacher Professional Identity Scale compiled by Wei et al. (2013). This study extracted core items reflecting professional belonging and value identity, constituting an 8-item brief version scale for administration.

*Job performance (P)*: adopted the scale developed by Williams and Anderson (1991) (14 items), covering task performance, contextual performance, etc., to evaluate teachers’ self-reported comprehensive work output over the past year.

*Turnover Intention (TI)*: adopted the 3-item scale developed by Mobley (1977) to measure the frequency and tendency of teachers to have recently developed thoughts of leaving the current organization or transferring from the school.

*Job burnout (JB)*: this study adopted a five-item brief version based on the Maslach burnout framework, with emotional exhaustion as the core content. The items were revised and validated in China by Li and Shi (2003), requiring teachers to answer based on the frequency of work exhaustion feelings (0 = Never, 4 = Always).

#### 3.3.3 Data analysis process and strategy

Statistical analyses were conducted using SPSS 26.0 and AMOS 26.0. Initial tests included reliability analysis, CFA, and Common Method Variance (CMV) checks via Harman’s single-factor test and an unmeasured latent common method factor (ULCMF) model.

Bootstrapping (5,000 resamples) estimated serial mediation and moderated mediation (Hayes, 2017). Due to low AVE in some constructs, the Heterotrait-Monotrait Ratio (HTMT) confirmed discriminant validity.

An item parceling strategy (Little et al., 2002) was applied for complex constructs (e.g., Differential Leadership) before CFA to improve parameter estimation stability while preserving theoretical dimensions.

Analyses control for demographic variables (gender, age, education). Terms such as “indirect effect” refer to statistical estimates; causal interpretations should be made with caution given the cross-sectional data.

## 4 Research results

To comprehensively examine the dual effect of differential leadership from the two complementary research contexts of “immediate situational response” and “long-term organizational ecology,” this section will sequentially report the situational experiment results of Study 1 and the field questionnaire survey results of Study 2, and conduct a comprehensive corroboration analysis of the design logic and data findings of the two studies at the end.

### 4.1 Study 1: scenario experiment data analysis

The core purpose of Study 1 was to experimentally examine whether relational-position cues (ingroup/outgroup) elicit different immediate psychological and behavioral responses among university teachers under controlled scenario conditions.

#### 4.1.1 Measurement quality and between-group homogeneity test

The results showed that the internal consistency coefficients of state social comparison (*α* = 0.747), state professional identity (*α* = 0.690), job performance tendency (*α* = 0.773), turnover intention tendency (*α* = 0.721), and job burnout tendency (*α* = 0.754) meet the requirements for scenario-adapted state measurement. Although the reliability of state professional identity was slightly below the conventional threshold of 0.70, it remained acceptable for a brief three-item scenario-adapted state measure.

To assess baseline equivalence between the two experimental groups, this study adopted a Chi-square (*χ*^2^) tests to assess whether the two experimental groups differed in baseline demographic characteristics. The results indicated that the “ingroup” and “outgroup” scenarios showed no significant differences in core demographic attributes such as gender (*χ*^2^ = 2.04, *p* = 0.361), age (*χ*^2^ = 2.80, *p* = 0.592), education (*χ*^2^ = 0.51, *p* = 0.775) title, post type, and years of teaching in universities. This indicates that the random assignment of data was effective, and the two groups of participants achieved a good homogeneity balance in baseline characteristics.

#### 4.1.2 Main effect test of differential position: immediate effect verification

Through independent samples *t*-tests, this study compared the psychological and behavioral reaction differences of teachers, as shown in Table 3. Additional ANCOVA models controlling for gender, age, and education yielded substantively identical conclusions. The manipulation check showed that participants in the two conditions differed significantly in perceived closeness and trust, indicating that the manipulation successfully induced the intended relational-position perception.

**Table 3:** Study 1 between-group difference test results.

Variable	Outgroup scenario M	Ingroup scenario M	*t*	*p*	Cohen’s d
Manipulation check	2.35	4.05	−20.29	<0.001	−1.48
State social comparison	2.85	2.28	8.40	<0.001	0.61
State professional identity	2.90	3.42	−7.28	<0.001	−0.53
Job performance tendency	2.87	3.51	−8.74	<0.001	−0.64
Turnover intention tendency	2.96	2.15	10.96	<0.001	0.80
Job burnout tendency	2.90	2.09	10.70	<0.001	0.78

Compared to the “ingroup” scenario, university teachers assigned to the “outgroup” scenario exhibited significantly heightened immediate state social comparison, diminished state professional identity, and weaker job performance tendency. As indicated in Table 3, all analytical effects reached medium to large effect sizes (*d > 0.5*).

These results suggest that relational-position cues are associated with clear position-dependent differences in teachers’ immediate psychological and behavioral responses. Specifically, outgroup cues trigger more intense comparison and withdrawal tendencies, whereas ingroup cues link to higher identity and performance. Thus, Study 1 provides experimental evidence consistent with the basic logic of H1, H2, and H3.

**Figure 2:**
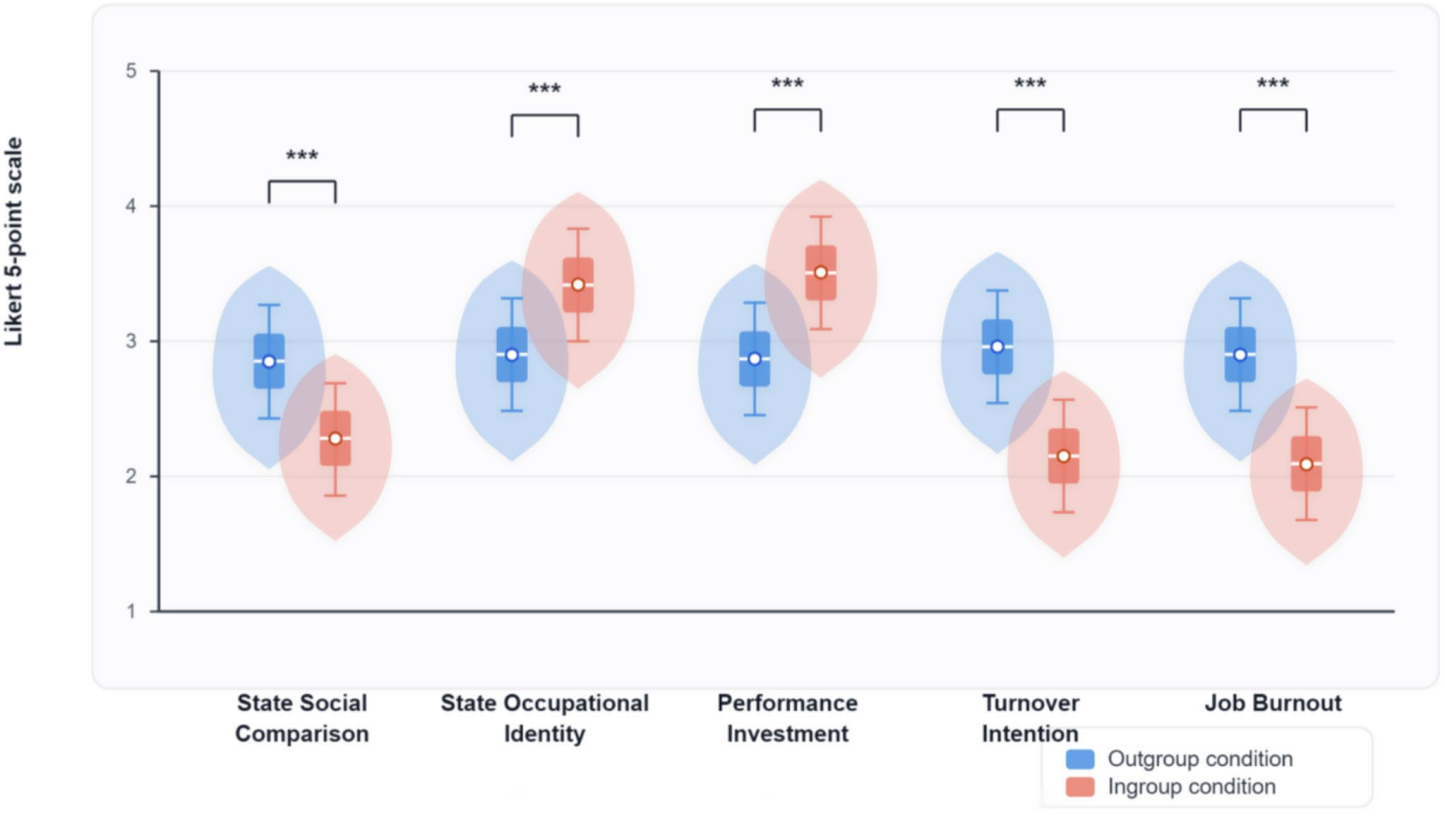
Comparison of teachers’ state psychological and behavioral tendencies under different relational-position conditions in Study 1. ***p < 0.001.

To present the size and direction of the experimental effects more intuitively, Figure 2 displays the distribution differences between the outgroup and ingroup scenarios on the five core state variables. The results indicate that participants assigned to the outgroup scenario exhibited significantly higher state social comparison, turnover intention tendencies, and job burnout tendency; in contrast, participants assigned to the ingroup scenario exhibited higher state professional identity and job performance tendency. The stable and obvious between-group separations across variables further corroborate the immediate psychological and behavioral consequences elicited by relational-position cues.

#### 4.1.3 State-based “serial transmission” and exploration of boundary conditions

To analyze the underlying psychological mechanism of the immediate experimental effect, this study further adopted the bootstrapping (resampling 5,000 times) to test the state-based “comparison—identity” mechanism in the experimental data. As shown in Table 4, the outgroup condition predicts significantly higher state social comparison than the ingroup condition. Higher social comparison was subsequently linked to lower state professional identity, which in turn predicted lower job performance tendency and higher turnover intention and job burnout. The serial indirect effects all remained significant, as their 95% confidence intervals did not include zero [for example, turnover intention tendency IE = 0.024, 95% CI (0.011, 0.038)]. This result, provides state-based experimental evidence supporting H4a, H4b, and H4c. However, these indirect effects should be interpreted as preliminary process evidence rather than definitive causal mediation due to the concurrent measurement of these variables.

**Table 4:** Bootstrapping test of state-based serial indirect effects in Study 1.

Influence path (under experimental state)	Indirect effect *B*	95% CI lower limit	95% CI upper limit	Conclusion
Outgroup condition → state SC → state OI → job performance tendency	−0.032	−0.050	−0.018	Significant
Outgroup condition → state SC → state OI → turnover intention tendency	0.024	0.011	0.038	Significant
Outgroup condition → state SC → state OI → job burnout tendency	0.025	0.013	0.042	Significant

In the serial indirect-effect analysis, the experimental condition contrast was specified as 0 = ingroup and 1 = outgroup.

A negative indirect effect for job performance tendency indicates that the outgroup condition results in lower job performance through intensified state social comparison and eroded state professional identity. Positive indirect effects indicate that the outgroup conditioncorresponds to stronger turnover and burnout-related tendencies through the same serial path.

In the exploratory interaction analysis of trait psychological capital, psychological capital had a significant main effect on lower state social comparison, whereas its interaction with the experimental condition was not significant. This suggests that, in immediate vignette context, psychological capital is insufficient to buffer the effect of outgroup relational-position cues on state social comparison.

To evaluate effect robustness, this study supplemented two exploratory analyses. First, a Two-way ANOVA tested the interaction between experimental grouping and subject characteristics. All interaction effects were insignificant (detailed in Table 5), reflecting that the immediate effects of outgroup relational-position cues were consistent across gender, age, and title groups. Second, the total effect of the outgroup condition on turnover intention was substantial *d = 0.80*, but the indirect transmission via “comparison → identity” only accounted for about 3.0%. In immediate vignette context, withdrawal intention may involve affective responses beyond the comparison–identity pathway; whereas the comparison–identity mechanism may become more evident as differential leadership experiences accumulate in long-term organizational ecologies, as examined in Study 2.

**Table 5:** Supplementary tests of interaction between experimental condition and demographic variables in Study 1.

Outcome variable	Interaction term examined	*F* value	Degrees of freedom	Significance (*p*)	Partial η^2^
State social comparison	Group × Title	0.01	(1, 748)	0.938	0.000
	Group × Age	0.01	(1, 748)	0.920	0.000
	Group × Gender	0.61	(1, 736)	0.434	0.001
State professional identity	Group × Title	0.09	(1, 748)	0.764	0.000
	Group × Age	0.00	(1, 748)	0.968	0.000
	Group × Gender	0.02	(1, 736)	0.888	0.000
Job performance tendency	Group × Title	0.74	(1, 748)	0.389	0.001
	Group × Age	0.03	(1, 748)	0.865	0.000
	Group × Gender	0.63	(1, 736)	0.428	0.001
Turnover intention tendency	Group × Title	2.98	(1, 748)	0.085	0.004
	Group × Age	0.10	(1, 748)	0.756	0.000
	Group × Gender	1.04	(1, 736)	0.308	0.001
Job burnout tendency	Group × Title	0.09	(1, 748)	0.770	0.000
	Group × Age	0.04	(1, 748)	0.842	0.000
	Group × Gender	0.77	(1, 736)	0.381	0.001

Interaction terms are constructed based on the experimental condition (Ingroup/Outgroup) and the respective demographic variables (high/low age groups, senior vs. junior/intermediate title groups, different genders). All interaction terms *p* > 0.05 and partial η^2^ are extremely small, indicating that the situational effect does not depend on specific demographic boundaries. Partial η^2^ is an effect size indicator.

### 4.2 Study 2: cross-sectional questionnaire data analysis

Study 2 examines naturally occurring organizational contexts and illustrates how teachers’ perceptions of differential leadership predict social comparison, professional identity, and workplace outcomes, and the boundary role of by psychological capital.

#### 4.2.1 Common method variance and reliability/validity checks

Given the cross-sectional self-reported data, common method variance (CMV) was evaluated using multiple diagnostic techniques, as recommended in prior methodological research on common method bias (Podsakoff et al., 2003). First, Harman’s single-factor test was conducted at the item level before item parceling by entering 65 individual items into an unrotated exploratory factor analysis. The result showed that the variance explained by the first common factor was 26.23%, below the 50% threshold.

To ensure the stability of the findings, an unmeasured latent common method factor (ULCMF) model was estimated to examine whether the substantive associations remained stable after accounting for method-induced covariance. As shown in Table 6, although the standardized coefficients decreased slightly after accounting for the latent method factor, all core path directions remained consistent and their statistical significance stayed robust. Specifically, the paths from differential leadership to social comparison, from social comparison to professional identity, and from professional identity to job performance, turnover intention, and job burnout showed no reversal in direction. These results suggest that common method variance is unlikely to explain the observed patterns, although CMV inflation cannot be completely ruled out.

**Table 6:** Comparison of core path coefficients before and after controlling for the unmeasured latent common method factor (Study 2).

Analytical path	Baseline *β*	ULCMF adjusted *β*	Direction consistency	Significance (*p*)
Differential leadership → social comparison	0.555	0.428	Consistent	< 0.001
Social comparison → professional identity	−0.468	−0.312	Consistent	< 0.001
Professional identity → job performance	0.491	0.385	Consistent	< 0.001
Professional identity → turnover intention	−0.441	−0.326	Consistent	< 0.001
Professional identity → job burnout	−0.454	−0.347	Consistent	< 0.001
Differential leadership → job burnout	0.479	0.362	Consistent	< 0.001

Subsequently, the Confirmatory Factor Analysis (CFA) results shown in Table 7 indicated that the seven-factor theoretical model had an excellent fit (*χ*^2^/df = 1.022, CFI = 0.998, TLI = 0.998, RMSEA = 0.006), and outperformed the alternative models. Notably, the single-factor model exhibited the poorest fit, further supporting the empirical distinctiveness of the constructs. Nevertheless, CMV remains a methodological limitation.

**Table 7:** CFA model fit comparison results.

Model	*χ* ^2^ */df*	CFI	TLI	RMSEA
Seven-factor model	1.022	0.998	0.998	0.006
Six-factor model (SC and OI merged)	1.460	0.952	0.950	0.025
Five-factor model (Outcome variables merged)	2.081	0.886	0.882	0.039
Single-factor model	4.875	0.590	0.577	0.074

#### 4.2.2 Normality test

Considering a large sample size (*N* = 713), this paper does not solely rely on the K-S test to judge whether the data are suitable for parametric analysis, but comprehensively judges by combining skewness, kurtosis, and the robustness of the Bootstrap method. As shown in Table 8, the data meet the basic requirements for subsequent regression and Bootstrap analyses.

**Table 8:** Normality test results of core variables.

Variable	Mean	SD	Skewness	Kurtosis	K-S statistic	*p*-value
Differential leadership (L)	2.899	0.889	0.067	−0.754	0.0470	0.083
Psychological capital (PC)	2.905	0.897	0.022	−0.787	0.0488	0.065
Social comparison (SC)	2.871	0.931	0.128	−0.781	0.0608	0.010
Professional identity (OI)	2.912	0.974	−0.016	−0.915	0.0722	0.001
Job performance (P)	2.893	0.991	0.038	−0.967	0.0564	0.021
Turnover intention (TI)	2.892	1.218	0.107	−1.184	0.1135	<0.001
Job burnout (BO)	1.881	1.122	0.119	−1.079	0.0909	<0.001

Under large sample conditions, a significant K-S test does not necessarily mean the data severely deviate from normality, so this study comprehensively judges combining skewness and kurtosis indicators.

#### 4.2.3 Descriptive statistics, reliability, validity, and correlation analysis

Descriptive statistics, reliability, convergent validity, and Pearson correlation coefficients are reported in Table 9.

**Table 9:** Descriptive statistics, reliability, validity, and correlation matrix.

Variable	M	SD	CR	AVE	1	2	3	4	5	6	7
1. Differential leadership (L)	2.90	0.89	0.920	0.434	**0.659**						
2. Psychological capital (PC)	2.90	0.90	0.913	0.466	−0.233**	**0.683**					
3. Social comparison (SC)	2.87	0.93	0.880	0.479	0.555**	−0.297**	**0.692**				
4. Professional identity (OI)	2.91	0.97	0.894	0.513	−0.465**	0.481**	−0.470**	**0.716**			
5. Job performance (P)	2.89	0.99	0.938	0.519	−0.393**	0.501**	−0.382**	0.495**	**0.720**		
6. Turnover intention (TI)	2.89	1.22	0.892	0.733	0.509**	−0.399**	0.494**	−0.440**	−0.335**	**0.856**	
7. Job burnout (BO)	1.88	1.12	0.900	0.644	0.479**	−0.363**	0.493**	−0.454**	−0.399**	0.394**	**0.802**

Bold values on the diagonal represent the square root of the Average Variance Extracted (AVE) for each construct. Off-diagonal values are the Pearson correlation coefficients. **p < 0.01.

In terms of reliability, the composite reliability (CR) values were greater than 0.880, indicating excellent internal consistency. Regarding convergent validity, the average variance extracted (AVE) values for professional identity, job performance, turnover intention, and job burnout exceeded the 0.50 threshold. It is noteworthy that the Average Variance Extracted (AVE) values for Differential Leadership, Psychological Capital, and Social Comparison (Table 9) were moderate. Given that the corresponding CR values were all above 0.88, these constructs were retained, although the measurement results should be interpreted with caution.

To examine discriminant validity, multiple criteria were considered. The seven-factor model showed excellent overall fit, and the square roots of the AVEs, shown as bold diagonal values in Table 9, were greater than the corresponding inter-construct correlations, satisfying the Fornell–Larcker criterion (Fornell and Larcker, 1981). In addition, all standardized factor loadings were statistically significant and ranged from 0.52 to 0.81.

The correlation analysis showed theoretically consistent relationships Differential leadership is positively predicts social comparison, turnover intention, and job burnout, while it is significantly negatively predicts professional identityand job performance. Social comparison was also negatively linked to professional identity, providing preliminary support for the subsequent mediation analyses.

To further assess discriminant validity in light of the moderate AVE values, the Heterotrait-Monotrait Ratio (HTMT) was calculated. As shown in Table 10, all HTMT values ranged from 0.259 to 0.634, below the conservative threshold of 0.85 (Henseler et al., 2015), providing additional support for the distinctiveness of the constructs.

**Table 10:** Heterotrait-Monotrait Ratio (HTMT) analysis results.

Variable	1. L	2. PC	3. SC	4. OI	5. P	6. TI	7. JB
1. Differential Leadership (L)	—						
2. Psychological Capital (PC)	0.259	—					
3. Social Comparison (SC)	0.634	0.342	—				
4. Professional Identity (OI)	0.525	0.548	0.551	—			
5. Job Performance (P)	0.429	0.550	0.431	0.553	—		
6. Turnover Intention (TI)	0.591	0.466	0.594	0.524	0.385	—	
7. Job Burnout (BO)	0.541	0.413	0.579	0.526	0.446	0.469	

L, Differential Leadership; PC, Psychological Capital; SC, Social Comparison; OI, Professional Identity; P, Job Performance; TI, Turnover Intention; BO, Job Burnout. HTMT values are absolute ratios and do not indicate the direction of associations. All values are below the conservative threshold of 0.85, supporting discriminant validity.

#### 4.2.4 Main associations and hierarchical regression analysis

To examine the direct associations between differential leadership and social comparison, professional identity, and workplace behavioral outcomes, and to evaluate its marginal explanatory power after excluding the influence of demographic variables (gender, age, education), this study constructed 6 independent hierarchical regression models, respectively. All models included control variables in the first block and their respective core predictor variables in the second block. The results are shown in Table 11.

**Table 11:** Hierarchical regression results of main associations and direct effects (Study 2).

Model	Outcome variable	Core predictor variable	*β*	*p*	*R* ^2^	ΔR^2^ (a)	*f* ^2^
Model 1	Social comparison (SC)	Differential leadership (L)	0.553	<0.001	0.310	—	—
Model 2	Professional identity (OI)	Social comparison (SC)	−0.468	<0.001	0.222	—	—
Model 3	Job performance (P)	Professional identity (OI)	0.491	<0.001	0.252	—	—
Model 4	Turnover intention (TI)	Professional identity (OI)	−0.441	<0.001	0.194	—	—
Model 5	Job burnout (BO)	Professional identity (OI)	−0.454	<0.001	0.206	—	—
Model 6	Job burnout (BO)	Differential leadership (L)	0.479	<0.001	0.231	0.228	0.297

(a) ΔR^2^ represents the increment in the model’s explanatory power after adding the core predictor variable on the basis of controlling demographic variables (gender, age, education). The ΔR^2^ and *f*^2^ values are specifically reported for Model 6 because this model directly evaluates the incremental explanatory power of differential leadership for job burnout. All models are independent hierarchical regression models, with control variables placed in the first block and respective core predictor variables in the second block. The β reported in the table is the standardized regression coefficient of the final model in the second block.

*Specifically*: After controlling for demographic attributes, the perception of differential leadership positively predicts social comparison, supporting H1; social comparison diminishes professional identity, supporting H2; professional identity positively relates to job performance, and negatively relates to turnover intention and job burnout, supporting H3a, H3b, and H3c.

In addition, Model 6 shows that the direct association of differential leadership on job burnout is significant. From the perspective of the change in model explanatory power, when only control variables are included, the *R*^2^ for job burnout is 0.003 (not listed in the table). After adding differential leadership, the *R*^2^ increases to 0.231, ΔR^2^ = 0.228, and the corresponding effect size *f*^2^ = 0.297, reaching a medium-to-strong level. This result indicates that in western universities, the perception of differential leadership is an important factor closely associated with teachers’ job burnout.

#### 4.2.5 Integrated mechanism test of serial mediation and boundary conditions

To examine the internal association mechanism linking differential leadership to teachers’ workplace outcomes and its boundary conditions, this section used the bootstrapping method with 5,000 resamples tested the serial indirect paths and the moderated serial mediation indices involving psychological capital.

As shown in Block I of Table 12, all paths involving social comparison and professional identity, were significant, as 95% confidence intervals did not include zero. The “social comparison–professional identity” mechanism (indirect effect = −0.185) indicates that differential leadership triggers higher social comparison, which further reduces professional identity and, subsequently, teachers’ workplace outcomes. Thus, H4a, H4b, and H4c were supported.

**Table 12:** Integrated test results of serial indirect effects and moderated indirect effects.

Test type	Path description	Effect size/index	95% CI	Conclusion
I. Basic mediation and serial mediation effects				
Simple/antecedent mediation	L → SC → OI	−0.185	[−0.236, −0.137]	Significant
Simple mediation	L → SC → BO	0.180	[0.126, 0.238]	Significant
Simple/subsequent mediation	L → OI → P	−0.119	[−0.162, −0.081]	Significant
Simple/subsequent mediation	L → OI → TI	0.078	[0.046, 0.116]	Significant
Simple/subsequent mediation	L → OI → BO	0.084	[0.053, 0.120]	Significant
Core serial mediation	L → SC → OI → P	−0.068	[−0.093, −0.047]	Significant
Core serial mediation	L → SC → OI → TI	0.045	[0.026, 0.066]	Significant
Core serial mediation	L → SC → OI → BO	0.048	[0.030, 0.069]	Significant
II. Moderating effect and moderated mediation effects				
Front-end moderating effect	L × PC → SC	−0.092	—	Significant
Moderated serial mediation	L → SC → OI → P	0.011	[0.002, 0.021]	Significant
Moderated serial mediation	L → SC → OI → TI	−0.007	[−0.015, −0.001]	Significant
Moderated serial mediation	L → SC → OI → BO	−0.008	[−0.015, −0.002]	Significant

L, Differential Leadership; PC, Psychological Capital; SC, Social Comparison; OI, Professional Identity; P, Job Performance; TI, Turnover Intention; BO. Job Burnout. The front-end moderating effect (L × PC) in the table reports the unstandardized regression coefficient (B), with test statistic *t* = −2.539, *p* = 0.011; the basic mediation model reports the indirect effect; the moderated mediation model reports the Index of Moderated Mediation. The 95% CI is derived based on 5,000 Bootstrapping resamples, and a confidence interval not containing 0 indicates a significant effect.

**Figure 3:**
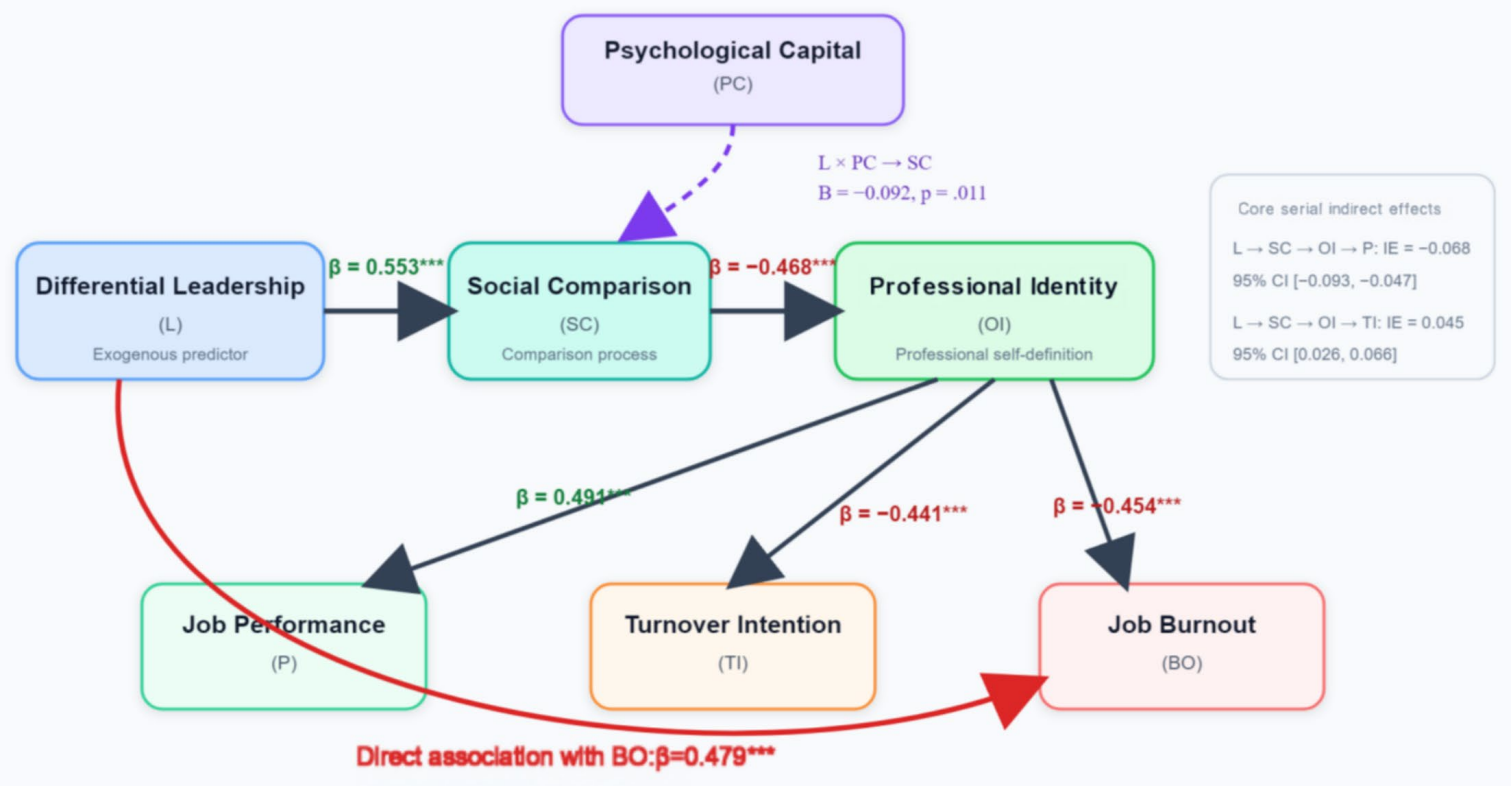
Mechanism path diagram linking differential leadership to teachers’ workplace outcomes. Standardized coefficients are displayed for direct paths. Indirect effects are based on 5,000 bootstrap resamples, and 95% confidence intervals excluding zero indicate significance. ***p < 0.001.

To present the internal association mechanism linking differential leadership to teachers’ workplace outcomes more intuitively, Figure 3 illustrates the key path relationships linking differential leadership to job performance, turnover intention, and job burnout through social comparison and professional identity. As depicted, differential leadership triggers social comparison, which subsequently erodes professional identity, ultimately predicting teachers’ behavioral and psychological outcomes.

**Figure 4:**
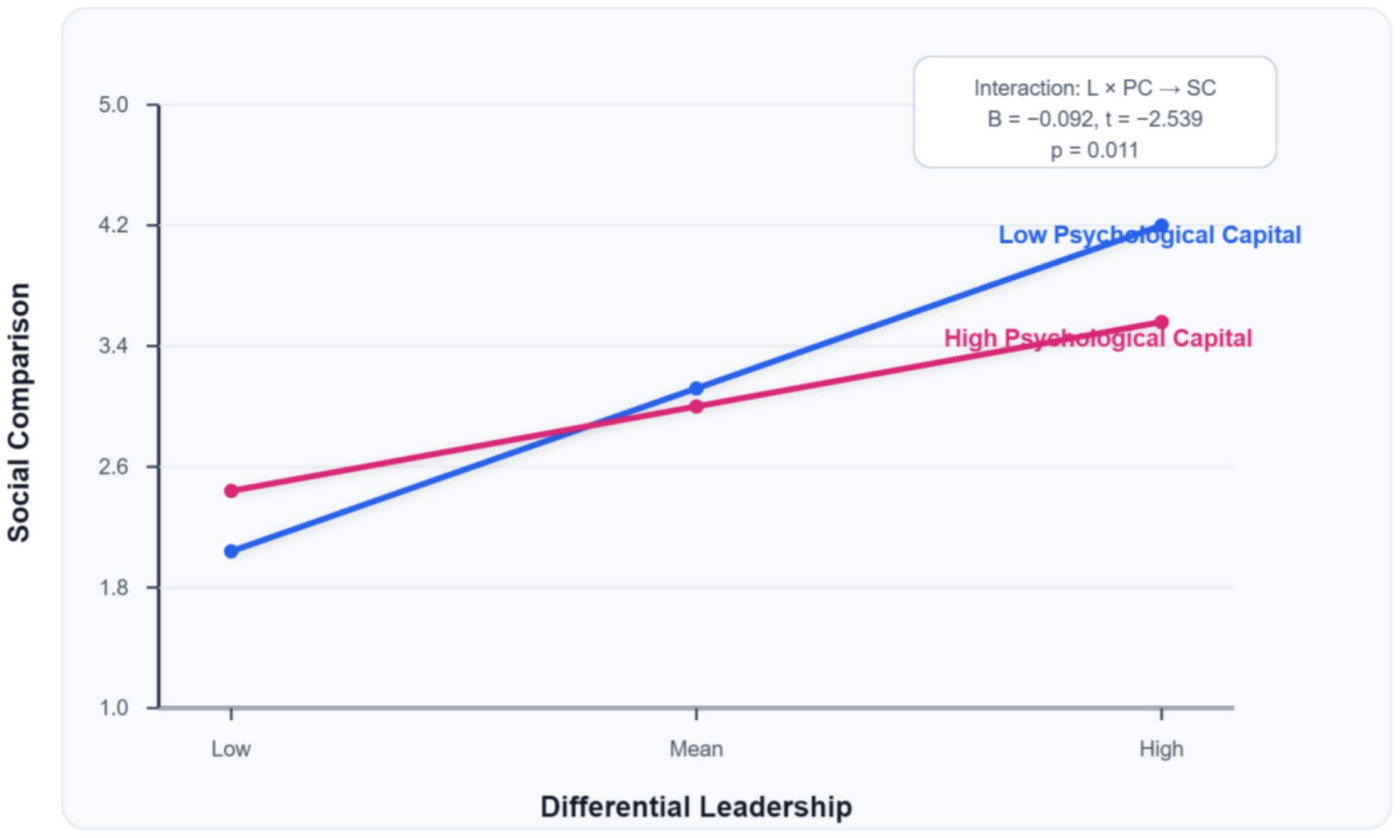
Psychological capital moderating effect diagram. The slope for high psychological capital is flatter than that for low psychological capital, indicating a buffering effect.

Based on this, the hierarchical analysis after controlling for demographic variables further showed that the interaction term “differential leadership × psychological capital” was significantly inversely related to social comparison. This indicates that higher psychological capital weakened the positive association between differential leadership and social comparison (see Figure 4). As shown in Block II of Table 12, across the three outcome pathways, the moderated serial mediation indices all reached significant levels. These results indicate that individuals’ psychological resources played a buffering role when teachers coped with perceived structural resource differences. Thus, H5 and H6 were supported.

Figure 4 plots the simple slopes of the relationship between differential leadership and social comparison under varying levels of psychological capital. Specifically, under conditions of high psychological capital, the positive impact of differential leadership on social comparison is significantly attenuated, demonstrating that psychological capital has an obvious buffering effect in situations of structural resource differences.

#### 4.2.6 Supplementary explanation of demographic differences and effect sizes

To avoid exaggerating minor statistical variations into meaningful group differences, this paper further reports effect sizes for demographic comparisons. As shown in Table 13, the difference in administrative duties on turnover intention did not reach a significant level, with its Cohen’s d = 0.084 and η^2^ = 0.0014, which belongs to an extremely small effect. This means that even if there are slight differences between different groups numerically, it is not enough to show that administrative identity has a substantive impact on the formation of turnover intention.

**Table 13:** Effect size results of demographic differences.

Analysis content	Statistic	*p*	Effect size	Interpretation
Administrative duties → turnover intention	*t* = 0.990	0.323	Cohen’s d = 0.084; η^2^ = 0.0014	Extremely small effect
Professional title → job burnout	*F* = 1.064	0.364	η^2^ = 0.0045	Extremely small effect
Differential leadership entering job burnout regression	*β* = 0.479	< 0.001	*f^2^* = 0.297	Medium-to-strong effect

The *p*-value in the third row of the table corresponds to the significance test result of the regression coefficient of differential leadership, and *f*^2^ is an effect size indicator, which itself does not independently correspond to a *p*-value.

Similarly, using professional title as a grouping variable to conduct a one-way ANOVA on job burnout, the result did not reach a significant level, with an effect size of η^2^ = 0.0045, which also belongs to a very small effect. In other words, although some demographic variables are often regarded as potential explanatory factors, their actual roles are far less potent than organizational relationship variables like differential leadership.

These findings are theoretically crucial, as they caution against attributing variations in teacher well-being solely to demographic attributes. Instead of assuming that age, professional title, or administrative status inherently determine a teacher’s psychological state, the data suggests that relational structures and perceived distribution experiences within the organization play a more decisive role.

### 4.3 Comprehensive corroboration analysis: a complete loop from “situational instinct” to “organizational ecology”

By synthesizing the results of both studies, this research provides complementary evidence regarding the associations between differential leadership and university teachers’ workplace outcomes across immediate situational responses and long-term organizational ecology, yielding two core theoretical insights:

First, the “double-edged sword” nature of differential leadership is closely bound to relational positions. While relying solely on cross-sectional surveys might suggest differential leadership is inherently detrimental—as the negative emotions of marginalized groups often dominate organizational climate evaluations—Study 1 underscores a different facet. When teachers are explicitly included in the resource “core circle,” this informal differential treatment may be interpreted as organizational trust, recognition, and developmental support, thereby fostering higher state professional identity and stronger job performance. Therefore, differential leadership is not a purely deleterious construct; whether it presents as support or pressure depends largely on the “near and far, close and distant” signals projected by management and the transparency of the rules underlying differentiated support.

Second, the “comparison—identity” composite mechanism showed convergent patterns across immediate and normalized contexts. Despite distinct methodologies, both studies consistently validate the theoretical relevance the of “differential perception → social comparison → professional identity → behavioral output” psychological chain. This finding has important implications for organizational behavior: In Study 1, the outgroup relational-position cue was accompanied by an immediate comparative reaction. In Study 2, this dynamic manifested as more stable associations among these variables in the real organizational context. In addition, the exploration of psychological capital context-dependent boundary conditions: sudden situational exclusion may temporarily weaken the individual’s defensive resources, rendering PC moderation non-significant in Study 1; whereas trait psychological capital may function as a stable buffering psychological resource, as reflected in Study 2.

Ultimately, this complementary dual-method design helps mitigate the attributional ambiguity inherent in single-method studies. It highlights the temporal and situational heterogeneity of differential leadership—linking relational cues to both immediate reactions and stable ecological perceptions. Nevertheless, given the differing designs and data structures, these synthesized findings offer suggestive insights rather than definitive proof of a longitudinal causal sequence.

## 5 Discussion

By integrating scenario experiments and cross-sectional surveys, this study explores the impact of differential leadership on the occupational status of university teachers in western China. This study contributes to the literature by unpacking the “double-edged sword” nature of differential leadership, validating the serial mediating roles of social comparison and professional identity, and identifying the contextual boundary of psychological capital.

### 5.1 Multiple effect characteristics of differential leadership

The findings underscore the dual and context-dependent nature of differential leadership. The scenario experiment (Study 1) shows that individuals in the “ingroup support” position, while perceiving resource and trust advantages, exhibit higher levels of state professional identity and job performance tendency; conversely, the field survey (Study 2) shows that in the normalized organizational ecology after controlling for demographic variables, structured differential perception predicts higher levels of social comparison, lower professional identity, and more negative workplace outcomes, including lower job performance, stronger turnover intention, and higher job burnout (Lu et al., 2025). This set of data mirrors the management reality of the university field: biased resource allocation has different decoding mechanisms for ingroup and outgroup members. In the absence of transparent rules, relatively closed differentiated support is easily attributed to relational bias. Based on this, differential leadership presents an organizational relationship attribute highly dependent on the situational position, rather than a monolithic management style, which is consistent with recent discussions on its double-edged effects (Liu N. et al., 2023; Liu Y. et al., 2023; Liu et al., 2026; Wang et al., 2025).

The positive responses observed in the ingroup condition can be understood as the socioemotional meaning attached to relational inclusion. When teachers perceive that leaders trust them, provide timely information, and offer developmental opportunities, differentiated treatment may be interpreted as a signal of support and relational recognition, thereby corresponding to higher state professional identity and stronger job performance tendency in the experimental scenario. This reinforces the notion that leadership dynamics are not merely about task allocation but are deeply rooted in the relational and psychological agency that drives employee engagement (Barattucci et al., 2020). However, this “light side” of differential leadership is conditional. If ingroup support is based primarily on opaque personal closeness rather than transparent developmental criteria, its positive meaning may coexist with comparison pressure and exclusion perceptions among outgroup members. Therefore, the implication is not to legitimize closed-circle favoritism, but to transform selective support into more transparent, rule-based, and development-oriented assistance.

### 5.2 The indirect association pattern of “social comparison—professional identity”

The research results validate the serial mediation of social comparison and professional identity between differential cues and teachers’ workplace outcomes, further confirming the critical link between professional identity and job burnout (Zhang et al., 2024). Moreover, current network-based perspectives emphasize the robust interplay between psychological empowerment and organizational identification, suggesting that the stabilization of a professional role is inextricably linked to the individual’s perceived psychological resources (Barattucci et al., 2025).

At the situational level, when individuals were assigned to the marginalized “outgroup” state social comparison surged instantaneously, accompanied by a decrease in state professional identity. This suggests that in the face of relational exclusion, comparison-based evaluation and a defensive retreat in self-worth appear to be an almost “instinctive response”; while in the normalized university research context (Study 2), due to the delay and uncertainty of work results, teachers are more likely to use external opportunity allocation and leader interaction frequency as important references to measure their own organizational value. At this time, the continuous comparison surrounding relational differences intertwines with long-term professional beliefs, and is accompanied by chronic burnout, emotional exhaustion, and a reduction in work engagement (Fang, 2025; Ramos Salazar et al., 2025).

This convergent pattern spanning “immediate situation (state variables)” and “macro ecology or real organizational context (trait tendencies)” indicates that social comparison is not an accidental emotional appendage, but a theoretically meaningful psychological link running through structural perception and terminal behavioral responses. The fluctuation of behavioral performance under the differential structure statistically mirrors the process in which teachers constantly calibrate their role value against resource distribution disparities.

### 5.3 Moderation and boundary heterogeneity of psychological capital

Psychological capital negatively moderates the positive association between differential leadership and social comparison, with this buffering effect extending statistically across the entire serial mediation path, aligning with its known protective role in professional settings (Zhang et al., 2024). For individuals with higher levels of psychological capital, the slope of their differential perception transforming into a social comparison is relatively flat.

The juxtaposition of the two studies suggests a possible “temporal heterogeneity” in this boundary effect. In the scenario experiment (Study 1), which simulated a sudden and acute relational exclusion, the buffering role of psychological capital was not significant. This finding does not imply that psychological capital is ineffective, but rather may point to a mechanism of ego depletion or the overwhelming of finite cognitive resources. As theories on self-regulation suggest, a sudden, intense negative event—such as being ostracized—can act as a high-intensity stimulus that instantaneously exhausts an individual’s cognitive resources for self-control. In this moment of acute shock, the defense mechanism provided by trait-based psychological capital may be “too slow” or “powerless” to activate. The individual’s reaction becomes more instinctual and automatic, temporarily overriding inherent psychological strengths and rendering individuals with varying levels of psychological capital equally vulnerable to the immediate sting of negative comparison.

In stark contrast, within the cross-sectional sample reflecting a long-term organizational ecology (Study 2), the moderating effect of psychological capital was statistically significant. This suggests that in a chronic, normalized environment of differential treatment, psychological capital may function less as an immediate shield and more as a sustainable reservoir of inner resources for adaptation and recovery. Individuals may have sufficient time to draw upon their hope, resilience, and optimism to cognitively reframe the persistent pressure, thereby maintaining psychological balance over the long run. It may serve as a key asset in a protracted battle of psychological attrition, steadily buffering the conversion of differential perceptions into corrosive social comparison.

This distinction provides a possible explanation for the operating conditions of psychological capital: it may serve as a potent buffer against chronic, predictable stressors, but its role under acute exclusion requires further verification.

### 5.4 Practical and management implications

Based on the above path associations, the results of this paper provide the following references for the support and governance practices of university teachers:

First, universities should enhance the transparency and interpretability of rules for differentiated resource allocation. Differentiated management has realistic value in expertise cultivation and project incubation, and its intervention focus should be placed on the openness of rules and the clarity of standards. Localized resource allocation lacking transparent procedures easily emits exclusion signals to relatively marginalized groups.

Second, pay attention to the organizational pressure generated by informal interactions. Outside of formal meetings and textual notifications, differences in information access and the frequency of symbolic interactions are important cues through which teachers judge their own relational positions, and managers should maintain a reasonable balance in daily communication.

Third, reduce unnecessary horizontal comparison within the organization. Considering the antecedent position of social comparison in the transmission of teaching anxiety and exhaustion (Ramos Salazar et al., 2025), management mechanisms should, in addition to outcome incentives, strengthen process feedback and project coaching, guide the evaluation system back to professional development itself, and downplay the relational attention induced by concentrated resource access.

Fourth, universities should stabilize professional identity through procedural construction. The effect of pure value advocacy on suppressing turnover intention is limited, as recent evidence emphasizes the complex mediating structures underlying retention and turnover (Feng et al., 2022; Ma et al., 2021). Teachers’ sustained focus on academic roles relies largely on an institutional ecology with consistent expectations, transparent procedures, institutional responsibility, and fairness perceptions.

### 5.5 Research limitations and future prospects

This study broadened the vision of mechanism argumentation through complementary internal and external validity, but there are still the following limitations that need to be further explored:

First, in terms of the scenario experiment (Study 1), although this text-reading method can better control extraneous interference and induce immediate psychological reactions, situational simulation still has ecological validity limitations compared to real organizational interactions. Participants did not face substantive resource deprivation and interest conflicts in the virtual scenario, and their self-reported engagement and withdrawal tendencies may be discounted compared to real workplace behaviors. In addition, the manipulation check relied on a single item assessing perceived relational closeness and trust. Although the very large between-group difference indicated that participants successfully perceived the intended ingroup–outgroup cue, a single-item check could not evaluate internal consistency reliability. Moreover, the salience of the two scenarios may have increased the possibility of demand characteristics. In addition, although responses with logical inconsistencies and failed manipulation checks were excluded, the study did not include a dedicated suspicion probe to assess whether participants guessed the experimental purpose. Therefore, demand characteristics cannot be fully ruled out. Future research should use multi-item manipulation checks, include realism and suspicion probes, and further manipulate other dimensions of differential leadership, such as partiality, care and communication, and tolerance for mistakes, to examine whether these dimensions produce similar immediate responses. Future studies may consider using field experiments or critical incident interviews for verification.

Second, regarding the field survey (Study 2), the cross-sectional data limits rigorous diachronic inferences. Future research should introduce the Experience Sampling Method (ESM) or a multi-wave cross-lagged design to observe the chronological characteristics of the dynamic evolution from perceived differential leadership to social comparison, professional identity, and workplace outcomes. In addition, Study 2 relied on single-source and single-occasion self-reported data. Although Harman’s single-factor test and the unmeasured latent common method factor diagnostic suggested that common method variance was unlikely to fully explain the findings, the possibility of inflated path coefficients cannot be completely ruled out. In particular, job performance was measured by self-report, which may be affected by social desirability and self-evaluation bias. Future studies should incorporate supervisor or peer ratings, objective indicators of teaching and research performance, and actual turnover data to further validate the model.

Third, the sample recruitment strategy may limit the generalizability of the findings. Study 1 relied on an online academic survey platform, and both studies were based on voluntary participation. Teachers who were more sensitive to fairness, resource allocation, or leader–member relationships may have been more willing to participate. In addition, the proportion of doctorate holders was relatively high, which may not fully represent the broader population of university teachers in western China. Future research should adopt more diversified sampling strategies and include teachers from different institutional types, disciplines, academic ranks, and regional contexts.

Fourth, the measurement of several constructs requires further refinement. The present study measured social comparison as a general comparison orientation. Although social comparison theory distinguishes upward, downward, and lateral comparison, the INCOM scale used in this study does not allow these directions to be separately modeled. Therefore, the absence of direction-specific comparison analysis is a measurement-driven limitation rather than an assumption that comparison directions are theoretically unimportant. Future research should employ direction-specific social comparison measures or experimental manipulations to examine whether upward, downward, and lateral comparisons exert different effects on teachers’ professional identity and workplace outcomes. In addition, the Average Variance Extracted (AVE) values for differential leadership, psychological capital, and social comparison were slightly below the ideal threshold of 0.50. Although the high Composite Reliability (CR), satisfactory CFA results, Fornell–Larcker evidence, and HTMT results support the overall measurement quality, the measurement results should still be interpreted cautiously, and the observed path coefficients may be affected by attenuation bias. Future studies should further contextualize the differential leadership scale for university settings and examine the internal structure of multidimensional constructs using second-order or bifactor models.

Fifth, this study did not fully test competing psychological mechanisms or cross-level boundary conditions. In terms of mechanism exclusion tests and cross-level association argumentation, the model setting of this study still has room for further exploration. For one, because the scenario experiment questionnaire focused on refining and capturing core constructs, we failed to parallelly collect immediate state emotions (such as state anger) or organizational justice perceptions, which means that this study could not test competing psychological mechanisms in parallel. For another, the moderated mediation effects relied on non-parametric Bootstrap tests for approximate estimation and did not adopt Hierarchical Linear Modeling (HLM) to coordinate the organizational climate variables of the school-level environment. Future research can introduce the regional resource abundance of higher education or differences in university types as cross-level moderating variables (Liu T. et al., 2024) to more meticulously analyze the interaction boundaries between macro institutions and micro relationships.

Sixth, the temporal heterogeneity of psychological capital should be interpreted cautiously. The difference between Study 1 and Study 2 was inferred from two different research designs rather than from a direct within-study manipulation of time scale. Therefore, the non-significant moderating effect of psychological capital in Study 1 and the significant moderating effect in Study 2 may reflect not only temporal differences but also differences in design, measurement context, measurement sensitivity, and data structure. The explanation that acute exclusion may temporarily overwhelm psychological capital should therefore be regarded as one possible interpretation rather than definitive evidence. Future longitudinal, experience-sampling, or repeated-scenario studies are needed to directly examine whether and how the buffering role of psychological capital changes under acute versus chronic differential-leadership conditions.

## 6 Conclusion

This study examined an integrated model linking differential leadership, teachers’ job performance, turnover intention, and job burnout in a sample of universities in western China. The results showed that differential leadership predicts heterogeneous reaction tendencies depending on individuals’ relational positions, and exerted negative workplace outcomes through the serial connection of social comparison and professional identity in a long-term structure. At the same time, psychological capital exerted a robust buffering effect against these pathogenic paths in the normalized organizational environment. Overall, differential bias is not a simple management style; as a relational structural element with signaling implications, it is closely related to the professional beliefs and continuous engagement of university teachers. Improving rule transparency and distribution predictability is a feasible path to reduce its negative organizational consequences.

## Data Availability

The original contributions presented in the study are included in the article/Supplementary material, further inquiries can be directed to the corresponding author.

## References

[ref1] Barattucci M. Lo Presti A. Bufalino G. Jønsson T. Teresi M. Pagliaro S. (2020). Distributed leadership agency and work outcomes: validation of the Italian DLA and its relations with commitment, trust, and satisfaction. Front. Psychol. 11:512. doi: 10.3389/fpsyg.2020.00512, 32296370 PMC7136843

[ref2] Barattucci M. Teresi M. Di Berardino C. Ramaci T. Pagliaro S. (2025). The relationship between psychological empowerment and organisational identification: a network analysis based approach. Int. J. Appl. Posit. Psychol. 10:35. doi: 10.1007/s41042-025-00229-x

[ref3] Cai L. Hu Y. Wu C. (2024). “Workers within the system”: a case study on the identity construction of local university teachers in the context of performance management. J. Educ. Stud. 20, 100–112. [in Chinese]. doi: 10.14082/j.cnki.1673-1298.2024.05.009

[ref4] Caliskan F. Idug Y. Uvet H. Gligor N. Kayaalp A. (2024). Social comparison theory: a review and future directions. Psychol. Mark. 41, 2823–2840. doi: 10.1002/mar.22087

[ref5] Cao X. Hangeldiyeva N. Ali A. Pitafi A. H. (2025). A social comparison perspective to investigating the influence of social media on employee creativity. Int. J. Hum. Comput. Interact. 13, 8481–8494. doi: 10.1080/10447318.2024.2410533

[ref6] Chen S. T. Haga K. Y. A. (2022). The influence of differential leadership and proactive personality on employee in-role performance: an integrated model. Front. Psychol. 13:978495. doi: 10.3389/fpsyg.2022.978495, 36619128 PMC9815459

[ref7] Fang A. (2025). The influence of teacher leadership on students academic burnout in China: potential multiple mediating effects of meeting basic psychological needs. Front. Psychol. 16:1558159. doi: 10.3389/fpsyg.2025.1558159, 40949351 PMC12424051

[ref8] Feng X. Wang Y. Jia P. Wang Y. Guan Z. Meng K. (2022). Associations between professional identity and turnover intent in prehospital emergency physicians: the mediating effect of burnout. Front. Public Health 10:1034925. doi: 10.3389/fpubh.2022.1034925, 36466526 PMC9713236

[ref9] Fornell C. Larcker D. F. (1981). Evaluating structural equation models with unobservable variables and measurement error. J. Mark. Res. 18, 39–50. doi: 10.1177/002224378101800104

[ref10] Gibbons F. X. Buunk B. P. (1999). Individual differences in social comparison: development of a scale of social comparison orientation. J. Pers. Soc. Psychol. 76, 129–142. doi: 10.1037//0022-3514.76.1.129, 9972558

[ref11] Halal Orfali C. Arancibia Muñoz M. L. Riquelme Plaza I. Unda Valenzuela R. (2024). How Higher Education Teachers see their Professional Identity. Front. Educ. 9:1429847. doi: 10.3389/feduc.2024.1429847

[ref12] Hayes A. F. (2017). Introduction to Mediation, Moderation, and Conditional Process Analysis: a Regression-based Approach. New York, NY: Guilford publications.

[ref13] Hazan-Liran B. Karni-Vizer N. (2024). Psychological capital as a mediator of job satisfaction and burnout among teachers in special and standard education. Eur. J. Spec. Needs Educ. 39, 337–351. doi: 10.1080/08856257.2023.2215009

[ref14] Henseler J. Ringle C. M. Sarstedt M. (2015). A new criterion for assessing discriminant validity in variance-based structural equation modeling. J. Acad. Mark. Sci. 43, 115–135. doi: 10.1007/s11747-014-0403-8

[ref15] Jiang H. Ye H. Zhang L. Wang X. (2023). The impact of differential leadership on employee performance: a moderated mediation model. J. Psychol. Sci. 21:705. [in Chinese]. doi: 10.12139/j.1672-0628.2023.05.018

[ref16] Jiang D. Zhang W. (2010). Chinese differential leadership and subordinate effectiveness. Indig. Psychol. Res. Chin. Soc. 34, 109–177. [in Chinese]. doi: 10.6254/IPRCS.201006_(33).0003

[ref17] Li C. Shi K. (2003). The influence of distributive justice and procedural justice on job burnout. Acta Psychol. Sin. 35, 677–684. [in Chinese]

[ref18] Little T. D. Cunningham W. A. Shahar G. Widaman K. F. (2002). To parcel or not to parcel: exploring the question, weighing the merits. Struct. Equ. Model. 9, 151–173. doi: 10.1207/S15328007SEM0902_1

[ref19] Liu Y. Houghton J. D. Chen S. Sun Y. Li D. Zou W. (2026). Differential leadership, employee dynamic capabilities, and innovative behavior: employees’ power distance orientation matters. Balt. J. Manag. 21, 36–56. doi: 10.1108/BJM-01-2025-0076

[ref20] Liu Y. Meng L. M. Wang H. Chen Y. (2024). Differential leadership and hospitality employees’ in-role performance: the role of constructive deviance and competitive climate. Int. J. Hosp. Manag. 122:103870. doi: 10.1016/j.ijhm.2024.103870

[ref21] Liu T. Wang J. Wang Y. (2024). Is a smaller pay gap always better? Empirical evidence based on an innovation perspective. Sci. Technol. Progr. Policy 41, 141–150. [in Chinese]. doi: 10.6049/kjjbydc.2023020323

[ref22] Liu Y. Zhang Z. Zhao H. Liu L. (2023). The double-edged sword effects of differential leadership on deviant behavior. Curr. Psychol. 42, 27888–27900. doi: 10.1007/s12144-022-03845-x, 36345549 PMC9631585

[ref23] Liu N. Zhang H. Zhou J. (2023). Unraveling the effect of differential leadership on employee performance: evidence from China. Front. Psychol. 14:1081073. doi: 10.3389/fpsyg.2023.1081073, 36935973 PMC10017960

[ref24] Lu J. Yang T. Qin Y. Hu X. (2025). Game-theoretic analysis of the impact of differential leadership on employee silence behavior in Chinese family enterprises. Front. Phys. 12:1536325. doi: 10.3389/fphy.2024.1536325

[ref25] Luthans F. Avolio B. J. Avey J. B. Norman S. M. (2007). Positive psychological capital: measurement and relationship with performance and satisfaction. Pers. Psychol. 60, 541–572. doi: 10.1111/j.1744-6570.2007.00083.x

[ref26] Ma L. Zhou F. Liu H. (2021). Relationship between psychological empowerment and the retention intention of kindergarten teachers: a chain intermediary effect analysis. Front. Psychol. 12:601992. doi: 10.3389/fpsyg.2021.601992, 33679521 PMC7928276

[ref27] Maslach C. Jackson S. (1981). The measurement of experienced burnout. J. Organ. Behav. 2, 99–113. doi: 10.1002/job.4030020205

[ref28] Maslach C. Schaufeli W. B. Leiter M. P. (2001). Job burnout. Annu. Rev. Psychol. 52, 397–422. doi: 10.1146/annurev.psych.52.1.397, 11148311

[ref29] Mobley W. H. (1977). Intermediate linkages in the relationship between job satisfaction and employee turnover. J. Appl. Psychol. 62:237. doi: 10.1037/0021-9010.62.2.237

[ref30] Podsakoff P. M. MacKenzie S. B. Lee J. Y. Podsakoff N. P. (2003). Common method biases in behavioral research: a critical review of the literature and recommended remedies. J. Appl. Psychol. 88, 879–903. doi: 10.1037/0021-9010.88.5.879, 14516251

[ref31] Qiu J. Shen H. (2025). The relationship between occupational adaptation and job burnout in special education teachers: the serial mediating effect of sense of control and psychological capital. Adv. Psychol. 15:1 [in Chinese]. doi: 10.12677/ap.2025.157395

[ref32] Ramos Salazar L. Garcia N. Huntington H. Brooks M. E. (2025). The mediating effects of social comparison on faculty burnout, teaching anxiety, and satisfaction among faculty who taught during the COVID-19 pandemic. Trends Psychol. 33, 1–22. doi: 10.1007/s43076-022-00246-8, 40479281 PMC9668215

[ref33] Uras Eren E. Atay D. (2025). Constructing professional identities: the role of school climate in early-career university teachers. Asian-Pac. J. Second. Foreign Lang. Educ. 10:22. doi: 10.1186/s40862-025-00329-w

[ref34] Wang S. Zhang W. Wang Z. Ma Y. Zhang C. (2025). Will differential leaders be welcomed? The justice-driven path to followership behavior. Curr. Psychol. 44, 18–32. doi: 10.1007/s12144-025-07392-z

[ref35] Wei S. Song G. Zhang D. (2013). The structure and scale of professional identity of primary and secondary school teachers in China. Teach. Educ. Res. 25, 55–60. [in Chinese]. doi: 10.13445/j.cnki.t.e.r.2013.01.007

[ref36] Williams L. J. Anderson S. E. (1991). Job satisfaction and organizational commitment as predictors of organizational citizenship and in-role behaviors. J. Manag. 17, 601–617. doi: 10.1177/014920639101700305, 18674448

[ref37] Xue D. Sun B. Li W. Zhou H. Ding F. Xiao W. (2023). The symptom network structure of teachers’ burnout and its connection to psychological capital. Psychol. Res. Behav. Manag. 16, 3503–3518. doi: 10.2147/PRBM.S421932, 37671266 PMC10476865

[ref38] You L. Li G. (2023). Overeducation and workers’ turnover intention. Foreign Econ. Manag. 45, 80–95 [in Chinese]. doi: 10.16538/j.cnki.fem.20230122.203

[ref39] Zhang Y. Li R. (2025). Linking psychological capital to the well-being of university teachers: the roles of work thriving, job burnout, and perceptions of politics. Work 81, 3272–3284. doi: 10.1177/10519815251330096, 40296769

[ref40] Zhang Q. Li W. Gao J. Sun B. Lin S. (2024). Teachers' professional identity and job burnout: the mediating roles of work engagement and psychological capital. Psychol. Schs. 61, 123–136. doi: 10.1002/pits.23039

