# Supplementary material for: From support to pressure: the multiple associated effects of differential leadership patterns on the workplace behavior of university teachers in western China
Source: Front Psychol. 2026 May 26;17:1855518. doi: 10.3389/fpsyg.2026.1855518 (PMC13246370; doi:10.3389/fpsyg.2026.1855518)
Supplement: Supplementary file 1 [file Data_Sheet_1.ZIP › Supplementary_Material_1_Codebook.docx]

**Variable Coding Scheme for Study 1 and Study 2**

**Table A1. Study 1 Codebook**

| Variable | Description | Coding |
| --- | --- | --- |
| id | Respondent ID | Numeric ID |
| study | Study indicator | 1 = Study 1 |
| condition | Experimental condition | 1 = Outsider, 2 = Insider |
| gender | Gender | 1 = Male, 2 = Female, 3 = Other/Prefer not to say |
| age | Age | 1 = <=30, 2 = 31-40, 3 = 41-50, 4 = 51-60, 5 = >60 |
| education | Highest education | 1 = Bachelor, 2 = Master, 3 = Doctoral |
| academic_rank | Academic rank | 1 = Teaching Assistant/Lecturer, 2 = Associate Professor, 3 = Professor, 4 = Other |
| position_type | Position type | 1 = Teaching & Research, 2 = Teaching-focused, 3 = Research-focused, 4 = Administrative |
| years_in_higher_education | Years in higher education | 1 = <=3 years, 2 = 4-10 years, 3 = 11-20 years, 4 = >=21 years |
| institution_type | Institution type | 1 = 985/211/Double First-Class, 2 = Provincial Key University, 3 = Regular University, 4 = Vocational College |
| province_region | Province/Region | 1 = Shaanxi, 2 = Gansu, 3 = Qinghai, 4 = Ningxia, 5 = Xinjiang, 6 = Sichuan, 7 = Chongqing, 8 = Guizhou, 9 = Yunnan, 10 = Tibet, 11 = Inner Mongolia, 12 = Guangxi |
| administrative_role | Administrative role | 0 = No, 1 = Yes |
| pc1-pc12 | Psychological capital items 1-12 | 1-5 |
| mc1 | Manipulation check | 1-5 |
| s_sc1-s_sc4 | State social comparison items 1-4 | 1-5 |
| s_oi1-s_oi3 | State professional identity items 1-3 | 1-5 |
| s_ti1-s_ti3 | Turnover intention items 1-3 | 1-5 |
| s_bo1-s_bo3 | Burnout/withdrawal intention items 1-3 | 1-5 |
| s_p1-s_p4 | Positive behavioral intention items 1-4 | 1-5 |

**Table A2. Study 2 Codebook**

| Variable | Description | Coding |
| --- | --- | --- |
| id | Respondent ID | Numeric ID |
| study | Study indicator | 2 = Study 2 |
| gender | Gender | 1 = Male, 2 = Female, 3 = Other/Prefer not to say |
| age | Age | 1 = <=30, 2 = 31-40, 3 = 41-50, 4 = 51-60, 5 = >60 |
| education | Highest education | 1 = Bachelor, 2 = Master, 3 = Doctoral |
| academic_rank | Academic rank | 1 = Teaching Assistant/Lecturer, 2 = Associate Professor, 3 = Professor, 4 = Other |
| position_type | Position type | 1 = Teaching & Research, 2 = Teaching-focused, 3 = Research-focused, 4 = Administrative |
| years_at_current_institution | Years at current institution | 1 = <=3 years, 2 = 4-10 years, 3 = 11-20 years, 4 = >=21 years |
| institution_type | Institution type | 1 = 985/211/Double First-Class, 2 = Provincial Key University, 3 = Regular University, 4 = Vocational College |
| province_region | Province/Region | 1 = Shaanxi, 2 = Gansu, 3 = Qinghai, 4 = Ningxia, 5 = Xinjiang, 6 = Sichuan, 7 = Chongqing, 8 = Guizhou, 9 = Yunnan, 10 = Tibet, 11 = Inner Mongolia, 12 = Guangxi |
| administrative_role | Administrative role | 0 = No, 1 = Yes |
| relationship_closeness | Relationship closeness with direct supervisor | 1 = Very close, 2 = Close, 3 = Neutral, 4 = Distant, 5 = Very distant |
| l1-l15 | Chaxu leadership items 1-15 | 1-5 |
| pc1-pc12 | Psychological capital items 1-12 | 1-5 |
| sc1-sc8 | Social comparison items 1-8 | 1-5 |
| oi1-oi8 | Professional identity items 1-8 | 1-5 |
| p1-p14 | Job performance items 1-14 | 1-5 |
| ti1-ti3 | Turnover intention items 1-3 | 1-5 |
| bo1-bo5 | Burnout items 1-5 | 0 = Never, 1 = Rarely, 2 = Sometimes, 3 = Often, 4 = Always |
