# Supplementary material for: From support to pressure: the multiple associated effects of differential leadership patterns on the workplace behavior of university teachers in western China
Source: Front Psychol. 2026 May 26;17:1855518. doi: 10.3389/fpsyg.2026.1855518 (PMC13246370; doi:10.3389/fpsyg.2026.1855518)
Supplement: Supplementary file 1 [file Data_Sheet_1.ZIP › Supplementary_Material_2_Instruments.docx]

# Supplementary Material

Research Instruments for a Multi-Study Investigation on Chaxu Leadership and Teacher Outcomes in Western Chinese Higher Education Institutions
西部高校差序领导与教师行为结果多子研究测量工具补充材料

**Editorial Note to Reviewers （致审稿人说明）**
This supplementary document contains the complete research instruments for two independent sub-studies (Study 1 and Study 2) that together constitute the multi-study design of this paper. The two studies were administered to two entirely separate and non-overlapping samples of university faculty members. Study 1 employed a between-subjects vignette experiment to establish causal relationships; Study 2 employed a cross-sectional field survey to establish external validity. Overlapping constructs (e.g., Psychological Capital) were measured independently in each study from their respective samples and serve different analytical roles within each study’s design.
本补充材料包含本文两个独立子研究（研究一与研究二）所使用的完整研究工具。两个子研究分别面向完全独立、互不重叠的高校教师样本施测。研究一采用组间情境实验设计，旨在建立变量间的因果关系；研究二采用横截面实地调查设计，旨在验证研究结论的外部效度。两个子研究中出现的重叠构念（如心理资本）均来自各自样本的独立测量，并在各自的研究设计中承担不同的分析功能。

## STUDY 1: Vignette Experiment

**研究一：情境实验**

**S1-Overview: Study Design and Procedure**
**研究一概述：研究设计与实施程序**
Study 1 employed a single-factor between-subjects experimental design with two conditions: Insider Status (insider condition) vs. Outsider Status (outsider condition). Participants were recruited from Credamo (见数), a professional academic survey platform in China, and were screened to include only currently employed faculty members at higher education institutions located in western China. Participants first completed baseline demographic information and a trait-level Psychological Capital measure. They were then randomly and automatically assigned by the survey platform to read one of two vignettes (Vignette A: Insider; Vignette B: Outsider) with equal probability (50%/50%). Following vignette exposure, participants completed manipulation check items, state-level mediator measures, and behavioral intention measures. The entire procedure took approximately 5 minutes.
研究一采用单因素组间实验设计，包含两个实验条件：核心圈（圈内人）条件与边缘圈（圈外人）条件。参与者通过中国专业学术调查平台"见数"（Credamo）招募，筛选条件为目前在职的中国西部高校教师。参与者首先完成基线人口学信息填写及特质层面的心理资本测量，随后由问卷平台以等概率（50%/50%）将其自动随机分配至两个情境之一（情境A：圈内人；情境B：圈外人）。情境阅读完毕后，参与者依次完成操纵检验题项、状态层面中介变量测量及行为意向测量。整个过程预计耗时约5分钟。

### S1-Part 1: Baseline Measures (Pre-Vignette)

**第一部分：基线测量（情境阅读前）**

**S1-1a: Demographic Information（人口学基本信息）**

| No. | Item（题项） | Response Options（选项） |
| --- | --- | --- |
| 1 | Gender 性别 | Male 男 / Female 女 / Prefer not to say 其他或不便说明 |
| 2 | Age 年龄 | ≤30 / 31–40 / 41–50 / 51–60 / >60 |
| 3 | Highest Education 最高学历 | Bachelor’s 学士 / Master’s 硕士 / Doctoral 博士 |
| 4 | Academic Rank 职称 | Teaching Assistant/Lecturer 助教/讲师 / Associate Professor 副教授 / Professor 教授 / Other 其他 |
| 5 | Position Type 岗位类型 | Teaching & Research 教学科研型 / Teaching-focused 教学为主型 / Research-focused 科研为主型 / Administrative 教学管理型 |
| 6 | Years in Higher Education 高校工作年限 | ≤3 years / 4–10 years / 11–20 years / ≥21 years |
| 7 | Institution Type 院校类型 | 985/211/Double First-Class 双一流 / Provincial Key University 省属重点本科 / Regular University 普通本科 / Vocational College 高职高专 |
| 8 | Province/Region 所在省份 | Shaanxi 陕西 / Gansu 甘肃 / Qinghai 青海 / Ningxia 宁夏 / Xinjiang 新疆 / Sichuan 四川 / Chongqing 重庆 / Guizhou 贵州 / Yunnan 云南 / Tibet 西藏 / Inner Mongolia 内蒙古 / Guangxi 广西 |
| 9 | Administrative Role 是否担任行政职务 | Yes 是 / No 否 |

**S1-1b: Baseline Psychological Capital Scale（基线心理资本量表）**
Scale Note（量表说明）： Measured on a 5-point Likert scale (1 = Strongly Disagree 完全不符合, 5 = Strongly Agree 完全符合). In Study 1, PsyCap was measured prior to vignette exposure as a baseline trait-level moderator to capture individual differences in dispositional psychological resources before experimental manipulation.
采用5点李克特计分（1=完全不符合，5=完全符合）。在研究一中，心理资本于情境阅读前测量，作为基线特质层面的调节变量，用以捕捉个体在实验操纵前的心理资源禀赋差异。

**Sub-dimension 1: Self-Efficacy 自我效能感**

| Item No. | Chinese Item 中文题项 | English Item 英文题项 |
| --- | --- | --- |
| PC1 | 即使工作环境不利，我也有信心完成自己的教学和科研目标 | Even in unfavorable work conditions, I am confident in achieving my teaching and research goals. |
| PC2 | 我相信自己有能力在工作中克服各种障碍和困难 | I believe I have the ability to overcome various obstacles and difficulties in my work. |
| PC3 | 我对自己在本职工作上的专业能力有充分信心 | I have full confidence in my professional competence in my current position. |

**Sub-dimension 2: Hope 希望**

| Item No. | Chinese Item 中文题项 | English Item 英文题项 |
| --- | --- | --- |
| PC4 | 即使遭遇工作挫折，我仍能找到多条路径实现自己的职业目标 | Even when facing setbacks, I can find multiple pathways to achieve my career goals. |
| PC5 | 目前我积极地为自己的职业发展设定目标并为之努力 | I am currently setting and actively pursuing goals for my career development. |
| PC6 | 我对自己未来的职业发展前景感到乐观 | I am optimistic about my future career development prospects. |

**Sub-dimension 3: Resilience 韧性**

| Item No. | Chinese Item 中文题项 | English Item 英文题项 |
| --- | --- | --- |
| PC7 | 当工作中遇到压力或挫折时，我能较快地恢复正常工作状态 | When facing work stress or setbacks, I can quickly recover to my normal work state. |
| PC8 | 我能从工作中的困难中学习并变得更加坚韧 | I can learn from work difficulties and become more resilient. |
| PC9 | 面对不公平的环境，我仍能保持积极的工作态度 | In the face of unfair circumstances, I can still maintain a positive work attitude. |

**Sub-dimension 4: Optimism 乐观**

| Item No. | Chinese Item 中文题项 | English Item 英文题项 |
| --- | --- | --- |
| PC10 | 对于工作中的不确定性，我倾向于从积极的角度看待 | I tend to look at work-related uncertainties from a positive perspective. |
| PC11 | 即使当前处境不佳，我也相信未来会有所改善 | Even when my current situation is difficult, I believe things will improve in the future. |
| PC12 | 我相信工作中的挑战最终会带来个人成长 | I believe that challenges at work will ultimately lead to personal growth. |

### **S1-Part 2: Experimental Vignettes（实验情境材料）**

Procedure Note（程序说明）： Participants were randomly assigned by the survey platform with equal probability (50%/50%) to read either Vignette A (Insider Condition) or Vignette B (Outsider Condition). Each participant was exposed to one vignette only. The vignettes were constructed to reflect realistic and culturally authentic scenarios of differential leader–member treatment in Chinese higher education institutions, based on the theoretical framework of Chaxu (differential order) leadership.
参与者由问卷平台以等概率（50%/50%）随机分配，只阅读情境A（圈内人条件）或情境B（圈外人条件）之一。两个情境的构建基于差序领导理论框架，旨在真实反映中国高校情境下领导对不同成员差异化对待的典型场景。

**Vignette A — Insider Condition（情境A：核心圈条件）**
Chinese Version（中文版）： “您目前在西部某本科院校任教。学院的张院长在管理中比较看重人际关系，对待不同教师在资源分配和制度执行上存在一定差异。在近半年的工作中，您明显感觉到自己属于张院长较为亲近和信任的群体。张院长经常私下与您交流学院的信息；在近期的重点课题申报中，他主动将唯一的重点推荐名额给了您；前段时间您因特殊原因导致某项教学材料迟交，张院长私下向您表示了理解，并未扣除您的绩效。您在工作中感受到了较多的关照与支持。”
English Version（英文版）： “You are currently a faculty member at a university in western China. The dean of your school, Director Zhang, places considerable emphasis on personal relationships in his management style and treats different faculty members differently in terms of resource allocation and rule enforcement. Over the past six months, you have clearly sensed that you belong to the group of faculty members whom Director Zhang trusts and treats with closeness. Director Zhang frequently communicates internal institutional information with you privately; in the recent key research grant nomination process, he proactively allocated the only priority recommendation slot to you; when you recently submitted certain teaching materials late due to special circumstances, Director Zhang privately expressed his understanding and did not deduct your performance bonus. You feel that you have received considerable care and support from your leader at work.”

**Vignette B — Outsider Condition（情境B：边缘圈条件）**
Chinese Version（中文版）： “您目前在西部某本科院校任教。学院的张院长在管理中比较看重人际关系，对待不同教师在资源分配和制度执行上存在一定差异。在近半年的工作中，您明显感觉到自己属于张院长较为疏远和边缘的群体。他与您的交流仅限于正式工作，缺乏私下沟通；在近期的重点课题申报中，尽管您的前期基础较好，但他依然将推荐名额给了另一位与他私交更好的教师；前段时间您因特殊原因导致某项教学材料迟交，张院长在工作群里严厉批评了您，并严格扣除了您的相关绩效。您在工作中感受到了明显的区别对待。”
English Version（英文版）： “You are currently a faculty member at a university in western China. The dean of your school, Director Zhang, places considerable emphasis on personal relationships in his management style and treats different faculty members differently in terms of resource allocation and rule enforcement. Over the past six months, you have clearly sensed that you belong to the group of faculty members whom Director Zhang keeps at a distance and treats as peripheral. His communication with you is limited to formal work occasions, with no private interaction; in the recent key research grant nomination process, despite your stronger preliminary research foundation, he allocated the recommendation slot to another faculty member with whom he has a closer personal relationship; when you recently submitted certain teaching materials late due to special circumstances, Director Zhang publicly criticized you in the department group chat and strictly deducted your full performance bonus. You feel that you have experienced obvious differential treatment at work.”

### S1-Part 3: Post-Vignette Measures（情境阅读后测量）

General Instruction（总填写说明）： The following items ask about your feelings and behavioral intentions assuming you are the protagonist in the scenario you just read. Please respond based on your honest first reaction. All items were rated on a 5-point Likert scale unless otherwise noted.
以下题项询问您假设自己处于刚才所阅读情境中的感受与行为意向，请根据您的真实第一反应作答。除特别说明外，所有题项均采用5点李克特计分。

**S1-3a: Manipulation Check（操纵检验）**
Scale: 1 = Strongly Disagree 完全不符合 — 5 = Strongly Agree 完全符合

| Item No. | Chinese Item 中文题项 | English Item 英文题项 |
| --- | --- | --- |
| MC1 | 在上述情境中，我认为自己属于张院长比较亲近和信任的人 | In the scenario described above, I consider myself to be someone whom Director Zhang trusts and treats with closeness. |

**S1-3b: State Social Comparison（状态社会比较）**
Scale Note（量表说明）： Original general social comparison items were reworded to capture state-level comparison tendencies triggered by the experimental manipulation of differential leader treatment.
原量表中的一般性社会比较倾向题项被调整为反映差序领导操纵所触发的状态层面比较倾向。
Scale: 1 = Strongly Disagree 完全不符合 — 5 = Strongly Agree 完全符合

| Item No. | Chinese Item 中文题项 | English Item 英文题项 |
| --- | --- | --- |
| S-SC1 | 在这种情况下，我会忍不住去对比那些受到张院长不同对待的同事 | In this situation, I would find myself involuntarily comparing myself to colleagues who receive different treatment from Director Zhang. |
| S-SC2 | 看到同事获得的不同待遇，我会强烈反思自己或者感到心理不平衡 | Seeing the different treatment my colleagues receive, I would feel a strong urge to reflect on myself or feel a sense of psychological imbalance. |
| S-SC3 | 我会非常在意自己在该学院资源分配中是否处于不利位置 | I would be very concerned about whether I am in a disadvantaged position in the school’s resource allocation. |
| S-SC4 | 我会经常把自己的工作待遇与受张院长重视的同事进行比较 | I would frequently compare my work treatment with that of colleagues who receive more attention from Director Zhang. |

**S1-3c: State Professional Identity（状态职业认同）**
Scale Note（量表说明）： Items were reworded from trait-level to state-level to capture identity responses triggered by the vignette scenario.
将原特质层面题项调整为状态层面，以捕捉情境触发的职业认同反应。
Scale: 1 = Strongly Disagree 完全不符合 — 5 = Strongly Agree 完全符合

| Item No. | Chinese Item 中文题项 | English Item 英文题项 |
| --- | --- | --- |
| S-OI1 | 即使处于这种环境下，我依然对"高校教师"这一身份感到自豪 | Even in this kind of environment, I still feel proud of my identity as a “university faculty member.” |
| S-OI2 | 在这种情况下，我依然觉得高校教师这份工作是有价值和意义的 | In this situation, I still believe that the work of a university faculty member is valuable and meaningful. |
| S-OI3 | 这种环境不会动摇我作为一名高校教师的职业信念 | This kind of environment would not shake my professional conviction as a university faculty member. |

**S1-3d: Behavioral Intentions — Dark Outcomes（黑暗面行为意向）**
Scale Note（量表说明）： Items were reworded from actual behavior to future behavioral intention to fit the hypothetical vignette design.
将反映实际行为的措辞调整为未来行为意向，以适配情境假设设计。
Scale: 1 = Extremely Unlikely 完全不可能 — 5 = Extremely Likely 极有可能

| Item No. | Chinese Item 中文题项 | English Item 英文题项 |
| --- | --- | --- |
| S-TI1 | 我会利用业余时间修改简历，寻找跳槽到其他单位的机会 | I would spend my spare time updating my resume and looking for opportunities to leave for another institution. |
| S-TI2 | 我会经常考虑离开目前的这所学校 | I would frequently think about leaving this school. |
| S-TI3 | 如果有合适的机会，我愿意立刻离开现在的单位 | If a suitable opportunity arose, I would be willing to leave this institution immediately. |
| S-BO1 | 想象在这样的环境中长期工作，我会感到精疲力竭 | Imagining working in such an environment long-term, I would feel exhausted and burned out. |
| S-BO2 | 在这种情境下，我可能会对学院的公共事务不再关心，只管好自己的事 | In this situation, I might stop caring about the school’s collective affairs and focus only on my own responsibilities. |
| S-BO3 | 想象每天去上班前，我都会感到心理疲惫不堪 | Imagining going to work each day, I would feel mentally exhausted before I even began. |

**S1-3e: Behavioral Intentions — Light Outcomes（光明面行为意向）**
Scale Note（量表说明）： Items were reworded to behavioral intention format to fit the hypothetical vignette design.
量表措辞专门调整为行为意向形式，以适配情境假设设计。
Scale: 1 = Extremely Unlikely 完全不可能 — 5 = Extremely Likely 极有可能

| Item No. | Chinese Item 中文题项 | English Item 英文题项 |
| --- | --- | --- |
| S-P1 | 如果有同事在工作中遇到困难，我依然愿意主动提供帮助 | If a colleague encountered difficulties at work, I would still be willing to proactively offer help. |
| S-P2 | 即使没有额外报酬，我也愿意参与学院层面的集体建设活动 | Even without additional compensation, I would be willing to participate in school-level collective activities. |
| S-P3 | 我依然会积极配合领导推动学院的各项正规工作 | I would still actively cooperate with leadership to advance the school’s formal work initiatives. |
| S-P4 | 我依然会保持高标准，努力完成我的日常教学和科研考核任务 | I would still maintain high standards and strive to fulfill my daily teaching and research performance requirements. |

## STUDY 2: Cross-Sectional Field Survey

**研究二：横截面实地问卷调查**

**S2-Overview: Study Design and Procedure**
**研究二概述：研究设计与实施程序**
Study 2 employed a cross-sectional field survey design to examine the hypothesized relationships among Chaxu leadership perception, psychological capital, social comparison, professional identity, job performance, burnout, and turnover intention in a real-world sample of university faculty. Participants were recruited through purposive sampling via faculty networks at higher education institutions in western China and through the Credamo academic survey platform. All participants were entirely separate from and non-overlapping with the Study 1 sample. The survey was administered anonymously online and took approximately 10–15 minutes to complete.
研究二采用横截面实地调查设计，在真实的高校教师样本中检验差序领导感知、心理资本、社会比较、职业认同、工作绩效、职业倦怠与离职意愿之间的理论关系。参与者通过目的性抽样，经由中国西部高校教师网络及"见数"（Credamo）学术调查平台进行招募。所有参与者均与研究一样本完全独立、互不重叠。问卷以匿名方式在线施测，预计完成时间为10至15分钟。

### S2-Part 1: Demographic Information（基本信息）

| No. | Item 题项 | Response Options 选项 |
| --- | --- | --- |
| 1 | Gender 性别 | Male 男 / Female 女 / Prefer not to say 其他或不便说明 |
| 2 | Age 年龄 | ≤30 / 31–40 / 41–50 / 51–60 / >60 |
| 3 | Highest Education 最高学历 | Bachelor’s 学士 / Master’s 硕士 / Doctoral 博士 |
| 4 | Academic Rank 职称 | Teaching Assistant/Lecturer 助教/讲师 / Associate Professor 副教授 / Professor 教授 / Other 其他 |
| 5 | Position Type 岗位类型 | Teaching & Research 教学科研型 / Teaching-focused 教学为主型 / Research-focused 科研为主型 / Administrative 教学管理型 |
| 6 | Years at Current Institution 在本校工作年限 | ≤3 years / 4–10 years / 11–20 years / ≥21 years |
| 7 | Institution Type 院校类型 | 985/211/Double First-Class 双一流 / Provincial Key University 省属重点本科 / Regular University 普通本科 / Vocational College 高职高专 |
| 8 | Province/Region 所在省份 | Shaanxi 陕西 / Gansu 甘肃 / Qinghai 青海 / Ningxia 宁夏 / Xinjiang 新疆 / Sichuan 四川 / Chongqing 重庆 / Guizhou 贵州 / Yunnan 云南 / Tibet 西藏 / Inner Mongolia 内蒙古 / Guangxi 广西 |
| 9 | Administrative Role 是否担任行政职务 | Yes 是 / No 否 |
| 10 | Perceived Relationship Closeness with Direct Supervisor 与直属领导关系的主观感知亲密程度 | Very close 非常亲密 / Close 较为亲密 / Neutral 一般/中等 / Distant 较为疏远 / Very distant 非常疏远 |

**S2-Part 2: Chaxu Leadership Perception Scale（差序领导行为感知量表）**
Scale Note（量表说明）： The scale was specifically contextualized for Chinese higher education institutions to measure faculty members’ perception of differential leadership behaviors across three dimensions: differential care, differential rule enforcement, and differential information sharing, plus overall insider/outsider position perception. All items were rated on a 5-point Likert scale (1 = Strongly Disagree 完全不符合, 5 = Strongly Agree 完全符合).
本量表用于测量教师对直属领导差序行为的感知，涵盖差异化关怀、差异化规则执行、差异化信息传递三个维度，以及圈层位置的整体感知。量表针对中国高校情境进行了专项情境化处理。采用5点李克特计分（1=完全不符合，5=完全符合）。

Sub-dimension 1: Differential Care 差异化关怀

| Item No. | Chinese Item 中文题项 | English Item 英文题项 |
| --- | --- | --- |
| L1 | 我的领导会主动关心与自己关系亲近的教师的工作和生活 | My leader proactively shows concern for the work and personal life of faculty members with whom he/she has close relationships. |
| L2 | 与领导关系亲近的教师，更容易获得额外的资源支持（如课题经费、办公条件等） | Faculty members with closer relationships with the leader find it easier to obtain additional resource support (e.g., research funding, office conditions). |
| L3 | 领导在分配科研或进修机会时，会优先考虑与自己亲近的教师 | When allocating research or professional development opportunities, the leader gives priority to faculty members with whom he/she has closer relationships. |
| L4 | 领导会为关系亲近的教师提供更多的职业发展机会（如推荐人才项目、晋升机会） | The leader provides more career development opportunities (e.g., talent program recommendations, promotion opportunities) to faculty members with close relationships. |
| L5 | 领导在与关系亲近的教师沟通时，更加开放坦诚 | The leader communicates more openly and frankly with faculty members with whom he/she has close relationships. |

Sub-dimension 2: Differential Rule Enforcement 差异化规则执行

| Item No. | Chinese Item 中文题项 | English Item 英文题项 |
| --- | --- | --- |
| L6 | 我的领导对不同教师执行规章制度的松紧程度不同 | My leader applies institutional rules and regulations with varying degrees of strictness toward different faculty members. |
| L7 | 与领导关系亲近的教师，在考核要求上会享有更大的弹性 | Faculty members with closer relationships with the leader enjoy greater flexibility in performance evaluation requirements. |
| L8 | 领导在奖惩决定中，会考虑教师与自己的私人关系 | The leader takes personal relationships with faculty members into account when making reward and punishment decisions. |
| L9 | 领导对部分与自己亲近的教师的工作失误更为宽容 | The leader is more tolerant of work mistakes made by faculty members with whom he/she has closer personal relationships. |

Sub-dimension 3: Differential Information Sharing 差异化信息传递

| Item No. | Chinese Item 中文题项 | English Item 英文题项 |
| --- | --- | --- |
| L10 | 领导会提前将重要信息告知与自己关系亲近的教师 | The leader shares important information in advance with faculty members with whom he/she has close relationships. |
| L11 | 我认为领导掌握的某些重要信息不会平等地传递给所有教师 | I believe that certain important information possessed by the leader is not equally shared with all faculty members. |
| L12 | 关系亲近的教师往往能获得更多非正式渠道的信息 | Faculty members with closer relationships tend to receive more information through informal channels. |

Sub-dimension 4: Insider/Outsider Position Perception 圈层感知

| Item No. | Chinese Item 中文题项 | English Item 英文题项 |
| --- | --- | --- |
| L13 | 我清楚地感知到自己在领导"亲疏圈子"中所处的位置 | I clearly sense my own position within the leader’s “closeness hierarchy.” |
| L14 | 我认为自己属于领导较为认可和信任的群体 | I consider myself to belong to the group of faculty members whom the leader recognizes and trusts more. |
| L15 | 我在工作中能感受到来自领导的区别对待 | I can feel that I am being treated differently from other colleagues by the leader in my work. |

**S2-Part 3: Psychological Capital Scale（心理资本量表）**
Scale Note（量表说明）： Measured on a 5-point Likert scale (1 = Strongly Disagree 完全不符合, 5 = Strongly Agree 完全符合). In Study 2, PsyCap was measured as a stable dispositional moderator reflecting participants’ real-world psychological resource levels in their actual workplace context, independent of any experimental manipulation.
采用5点李克特计分（1=完全不符合，5=完全符合）。在研究二中，心理资本作为稳定人格特质调节变量，反映参与者在真实工作情境中的实际心理资源水平，独立于任何实验操纵之外。

Sub-dimension 1: Self-Efficacy 自我效能感

| Item No. | Chinese Item 中文题项 | English Item 英文题项 |
| --- | --- | --- |
| PC1 | 即使工作环境不利，我也有信心完成自己的教学和科研目标 | Even in unfavorable work conditions, I am confident in achieving my teaching and research goals. |
| PC2 | 我相信自己有能力在工作中克服各种障碍和困难 | I believe I have the ability to overcome various obstacles and difficulties in my work. |
| PC3 | 我对自己在本职工作上的专业能力有充分信心 | I have full confidence in my professional competence in my current position. |

**Sub-dimension 2: Hope 希望**

| Item No. | Chinese Item 中文题项 | English Item 英文题项 |
| --- | --- | --- |
| PC4 | 即使遭遇工作挫折，我仍能找到多条路径实现自己的职业目标 | Even when facing setbacks, I can find multiple pathways to achieve my career goals. |
| PC5 | 目前我积极地为自己的职业发展设定目标并为之努力 | I am currently setting and actively pursuing goals for my career development. |
| PC6 | 我对自己未来的职业发展前景感到乐观 | I am optimistic about my future career development prospects. |

**Sub-dimension 3: Resilience 韧性**

| Item No. | Chinese Item 中文题项 | English Item 英文题项 |
| --- | --- | --- |
| PC7 | 当工作中遇到压力或挫折时，我能较快地恢复正常工作状态 | When facing work stress or setbacks, I can quickly recover to my normal work state. |
| PC8 | 我能从工作中的困难中学习并变得更加坚韧 | I can learn from work difficulties and become more resilient. |
| PC9 | 面对不理想的管理环境，我仍能保持积极的工作态度 | In the face of an unfavorable management environment, I can still maintain a positive work attitude. |

**Sub-dimension 4: Optimism 乐观**

| Item No. | Chinese Item 中文题项 | English Item 英文题项 |
| --- | --- | --- |
| PC10 | 对于工作中的不确定性，我倾向于从积极的角度看待 | I tend to look at work-related uncertainties from a positive perspective. |
| PC11 | 即使当前处境不佳，我也相信未来会有所改善 | Even when my current situation is difficult, I believe things will improve in the future. |
| PC12 | 我相信工作中的挑战最终会带来个人成长 | I believe that challenges at work will ultimately lead to personal growth. |

**S2-Part 4: Social Comparison Scale（社会比较量表）**
Scale Note（量表说明）： Original items were reworded to reflect the specific context of differential leader–member resource distribution in Chinese higher education institutions. The adapted items retain the core psychological construct of social comparison orientation while enhancing ecological validity for the target population. All items rated on a 5-point Likert scale (1 = Strongly Disagree 完全不符合, 5 = Strongly Agree 完全符合).
调整为反映中国高校情境下差序领导资源分配的具体情境，在保留社会比较核心心理构念的同时，提升了对目标群体的生态效度。采用5点李克特计分。

Sub-dimension 1: Upward Social Comparison 上行社会比较

| Item No. | Chinese Item 中文题项 | English Item 英文题项 |
| --- | --- | --- |
| SC1 | 我常常关注那些比我获得更多领导支持的同事，并思考差距在哪里 | I often pay attention to colleagues who receive more support from the leader than I do and think about where the gap lies. |
| SC2 | 看到同事获得领导的资源倾斜，我会反思自己哪里做得不够好 | When I see colleagues receiving preferential resource allocation from the leader, I reflect on where I might be falling short. |
| SC3 | 与获得更多机会的同事比较，激励我更加努力地工作 | Comparing myself with colleagues who receive more opportunities motivates me to work harder. |
| SC4 | 我会将与领导关系更好的同事作为自己努力的参照标准 | I use colleagues who have better relationships with the leader as a reference standard for my own efforts. |

**Sub-dimension 2: Downward Social Comparison 下行社会比较**

| Item No. | Chinese Item 中文题项 | English Item 英文题项 |
| --- | --- | --- |
| SC5 | 与得到领导关注较少的同事比较，让我感到自己还不错 | Comparing myself with colleagues who receive less attention from the leader makes me feel that I am doing alright. |
| SC6 | 在感到工作压力时，我会想到还有同事处境比我更困难 | When feeling work pressure, I remind myself that there are colleagues who are in more difficult situations than I am. |

**Sub-dimension 3: Comparison Frequency and Tendency 比较频率与倾向**

| Item No. | Chinese Item 中文题项 | English Item 英文题项 |
| --- | --- | --- |
| SC7 | 我经常会把自己的工作待遇与同事进行比较 | I frequently compare my work treatment and conditions with those of my colleagues. |
| SC8 | 当领导对不同教师差别对待时，我会特别在意自己是否处于不利位置 | When the leader treats different faculty members differently, I pay particular attention to whether I am in a disadvantaged position. |

**S2-Part 5: Professional Identity Scale（职业认同量表）**
Scale Note（量表说明）： Items cover professional belonging, organizational identification, and cognitive evaluation of the differential leadership context. All items rated on a 5-point Likert scale (1 = Strongly Disagree 完全不符合, 5 = Strongly Agree 完全符合).
涵盖职业归属感、组织认同感及对差序文化的认知评价三个方面。采用5点李克特计分。

**Sub-dimension 1: Professional Belonging 职业归属感**

| Item No. | Chinese Item 中文题项 | English Item 英文题项 |
| --- | --- | --- |
| OI1 | 我对"高校教师"这一职业身份感到自豪 | I feel proud of my professional identity as a “university faculty member.” |
| OI2 | 我认为高校教师是一个值得长期投入的职业 | I believe that being a university faculty member is a profession worth long-term commitment. |
| OI3 | 我愿意向他人表明自己是一名高校教师 | I am willing to tell others that I am a university faculty member. |

**Sub-dimension 2: Organizational Identification 组织认同感**

| Item No. | Chinese Item 中文题项 | English Item 英文题项 |
| --- | --- | --- |
| OI4 | 我认为自己所在学院的成功就是我的成功 | I see the success of my school as my own success. |
| OI5 | 当有人批评我所在的学院时，我会感到不舒服 | When someone criticizes my school, I feel uncomfortable. |
| OI6 | 我对学院的发展前景感到认同和期待 | I feel a sense of identification with and anticipation for the development prospects of my school. |

**Sub-dimension 3: Cognitive Evaluation of Differential Leadership Context 对差序文化的认知评价**

| Item No. | Chinese Item 中文题项 | English Item 英文题项 |
| --- | --- | --- |
| OI7 | 我认为在高校中领导与教师之间存在亲疏差异是正常现象 | I believe it is normal for varying degrees of closeness to exist between leaders and faculty members in universities. |
| OI8 | 即使在组织中处于相对边缘的位置，我仍然认可自己的工作价值 | Even when I am in a relatively peripheral position within the organization, I still recognize the value of my work. |

**S2-Part 6: Job Performance Scale（工作绩效量表）**
Scale Note（量表说明）： Contextualized for Chinese higher education faculty. Items reflect teaching and research performance as task performance, helping and civic behaviors as contextual performance, and adaptability as adaptive performance. All items ask about behaviors over the past year and are rated on a 5-point Likert scale (1 = Strongly Disagree 完全不符合, 5 = Strongly Agree 完全符合).
针对中国高校教师情境进行了专项设计。题项分别反映教学科研绩效（任务绩效）、帮助行为与公民行为（关系绩效）及适应变化的能力（适应性绩效）。所有题项询问受访者过去一年的实际工作行为表现，采用5点李克特计分。

**Sub-dimension 1: Task Performance 任务绩效**

| Item No. | Chinese Item 中文题项 | English Item 英文题项 |
| --- | --- | --- |
| P1 | 我能高质量地完成既定的教学任务（备课、授课、辅导等） | I am able to complete my designated teaching tasks (lesson preparation, instruction, tutoring, etc.) with high quality. |
| P2 | 我能按时完成科研相关任务（发表论文、申报课题等） | I am able to complete research-related tasks (publishing papers, applying for grants, etc.) on time. |
| P3 | 我积极参与教学改革与课程建设工作 | I actively participate in teaching reform and curriculum development work. |
| P4 | 我的工作成果达到了学院规定的考核要求 | My work outcomes meet the performance evaluation requirements set by my school. |
| P5 | 我能有效地将教学与科研相结合，产出高质量成果 | I am able to effectively integrate teaching and research to produce high-quality outcomes. |

**Sub-dimension 2: Contextual Performance / Organizational Citizenship Behavior 关系绩效/组织公民行为**

| Item No. | Chinese Item 中文题项 | English Item 英文题项 |
| --- | --- | --- |
| P6 | 我愿意帮助有需要的同事解决工作中的问题 | I am willing to help colleagues in need solve problems at work. |
| P7 | 即使没有额外报酬，我也愿意参加学院组织的集体活动 | Even without additional compensation, I am willing to participate in collective activities organized by the school. |
| P8 | 我会主动维护学院和部门的良好形象 | I proactively maintain the positive image of my school and department. |
| P9 | 我乐于分享自己的教学和科研经验，促进团队共同成长 | I am happy to share my teaching and research experience to promote the collective growth of the team. |
| P10 | 我会积极配合领导推动学院各项工作 | I actively cooperate with leadership to advance various work initiatives in the school. |

**Sub-dimension 3: Adaptive Performance 适应性绩效**

| Item No. | Chinese Item 中文题项 | English Item 英文题项 |
| --- | --- | --- |
| P11 | 面对政策调整和考核标准变化，我能较快地适应和调整 | When facing policy adjustments and changes in evaluation standards, I can adapt and adjust relatively quickly. |
| P12 | 我能积极应对工作中的新挑战和不确定性 | I can actively respond to new challenges and uncertainties in my work. |
| P13 | 我善于学习新知识和新技能以提升工作能力 | I am good at learning new knowledge and skills to improve my work competence. |
| P14 | 我在工作遭遇挫折时能保持较好的心理承受力 | I am able to maintain good psychological resilience when encountering setbacks at work. |

**S2-Part 7: Turnover Intention Scale（离职意愿量表）**
Scale Note（量表说明）： All items rated on a 5-point Likert scale (1 = Strongly Disagree 完全不符合, 5 = Strongly Agree 完全符合).
采用5点李克特计分（1=完全不符合，5=完全符合）。

| Item No. | Chinese Item 中文题项 | English Item 英文题项 |
| --- | --- | --- |
| TI1 | 我经常考虑离开现在所在的高校 | I often think about leaving my current university. |
| TI2 | 我有可能在未来一年内离职或调离本校 | It is possible that I will resign or transfer away from this institution within the next year. |
| TI3 | 如果有合适的机会，我愿意跳槽到其他单位 | If a suitable opportunity arose, I would be willing to change jobs and move to another institution. |

**S2-Part 8: Burnout Scale（职业倦怠量表）**
Scale Note（量表说明）： Items cover emotional exhaustion, depersonalization, and reduced sense of personal accomplishment. Response format uses a 5-point frequency scale (0 = Never 从不, 1 = Rarely 偶尔, 2 = Sometimes 有时, 3 = Often 经常, 4 = Always 总是).
涵盖情绪耗竭、去人性化及个人成就感降低三个方面。采用频率计分格式（0=从不，1=偶尔，2=有时，3=经常，4=总是）。

| Item No. | Chinese Item 中文题项 | English Item 英文题项 |
| --- | --- | --- |
| BO1 | 我感到工作令我精疲力竭 | I feel emotionally drained by my work. |
| BO2 | 我对学生和同事的耐心越来越少，有时显得冷漠 | I am becoming less patient with students and colleagues, and sometimes appear indifferent. |
| BO3 | 我觉得自己在工作上的努力已无法发挥真正的价值 | I feel that my efforts at work can no longer produce meaningful value. |
| BO4 | 每天上班之前我都感到疲惫不堪 | I feel exhausted before I even start work each day. |
| BO5 | 我觉得自己的日常工作缺乏意义 | I feel that my daily work lacks meaning. |
